# Cumulative respiratory pathogen detection burden by multiplex PCR is associated with mortality in a TB-enriched ICU cohort

**DOI:** 10.1128/spectrum.00865-26

**Published:** 2026-07-22

**Authors:** Jurong Yan, Qian He, Lihui Zhao, Xunfei Yi, Aojia Wei, Ning Meng, Yi Ren

**Affiliations:** 1 Department of Clinical Laboratory, Wuhan Pulmonary Hospital 577900, Wuhan, China; 2 Guangzhou Biotron Technology Co., Ltd., Guangzhou, China; Beijing Institute of Genomics, Chinese Academy of Sciences, Beijing, China; Beijing Institute of Genomics, Chinese Academy of Sciences, Beijing, China; Beijing Chest Hospital, Capital Medical University, Beijing, China

**Keywords:** multiplex PCR, lower respiratory tract infections, intensive care unit, co-detection, pathogen detection burden, mortality

## Abstract

Rapid multiplex polymerase chain reaction (mPCR) is increasingly used in intensive care units (ICUs), but the prognostic value of cumulative pathogen detection burden in critically ill patients with suspected pulmonary tuberculosis (TB) or complex respiratory infections remains unclear. We retrospectively studied 130 ICU patients with severe respiratory symptoms and suspected TB or complex respiratory infections. Bronchoalveolar lavage fluid (BALF) was analyzed by mPCR within 48 h of admission. Multivariable logistic and Cox proportional hazards regression models evaluated the association between total pathogen detection count and mortality after adjustment for age, Acute Physiology and Chronic Health Evaluation II score, prior anti-infective therapy, and pre-ICU hospitalization duration. The mPCR positivity rate was 93.8%, with mixed-category co-detections in 46.9%. The most frequently detected targets were *Mycobacterium tuberculosis* (65.4%), *Candida albicans* (38.5%), and *Pseudomonas aeruginosa* (30.0%). Receiver operating characteristic analysis identified an exploratory threshold of >3 detected targets for mortality risk stratification. In adjusted models, total pathogen detection count remained associated with in-hospital mortality (adjusted OR 1.55, 95% CI 1.14–2.16; *P* = 0.007) and 60 day mortality hazard (adjusted HR 1.42, 95% CI 1.11–1.82; *P* = 0.006). Sensitivity analyses excluding *Candida albicans* or detections with raw Ct values >35 showed similar findings. In this single-center, TB-enriched ICU cohort, BALF mPCR revealed frequent mixed-category pathogen detections. Cumulative pathogen detection burden was associated with short-term mortality after adjustment for clinical severity and selected pre-sampling exposures. Because molecular detection does not necessarily indicate active infection, these findings should be interpreted as evidence that pathogen detection complexity may serve as an exploratory risk-stratification signal requiring prospective validation.

**IMPORTANCE:**

Rapid molecular assays frequently detect multiple respiratory pathogens in critically ill patients, but the clinical interpretation of these complex results remains challenging. In this single-center intensive care unit (ICU) cohort enriched for suspected tuberculosis and complex respiratory infections, bronchoalveolar lavage fluid multiplex polymerase chain reaction showed frequent mixed-category pathogen detections. A higher cumulative pathogen detection count was associated with increased short-term mortality after adjustment for illness severity, prior anti-infective therapy, and pre-ICU hospitalization duration. These findings suggest that pathogen detection complexity may provide a risk-stratification signal beyond simple pathogen identification. However, molecular detection does not necessarily indicate active infection, and the observed association should not be interpreted as evidence that reducing detection burden alone would improve outcomes. Prospective multicenter studies incorporating adjudicated infection status, antimicrobial exposure, host immune status, and semi-quantitative pathogen-load metrics are needed to validate whether this metric can be incorporated into ICU prognostic models.

## INTRODUCTION

Lower respiratory tract infections (LRIs) remain a leading cause of morbidity and mortality in intensive care units (ICUs) worldwide (1). In fact, recent global data indicate that LRIs were the most fatal infectious disease excluding COVID-19 in 2021, accounting for approximately 2.5 million deaths and exerting a profound public health burden (2). In the critical care setting, severe pneumonia and respiratory infections often deteriorate rapidly and may present with acute respiratory failure requiring mechanical ventilation or septic shock requiring vasopressors, thereby necessitating ICU-level organ support (3). Consequently, case fatality rates often reach 20% to 50%, depending on the patient population and overall illness severity (4). Given the urgency of initiating life-saving targeted therapies, delayed diagnosis and delayed pathogen identification may contribute to prolonged hospital stays and increased mortality risk (5, 6). This clinical dilemma reinforces a core principle emphasized by the Surviving Sepsis Campaign guidelines: clinicians must obtain appropriate routine microbiologic cultures prior to antimicrobial administration, strictly provided that doing so does not substantially delay vital treatment (7).

While conventional microbial culture remains the reference standard for diagnosis, its prolonged turnaround time and susceptibility to false-negative results following empirical antibiotic exposure often fail to meet the time-sensitive demands of the ICU (8, 9). To address these limitations, rapid multiplex polymerase chain reaction (mPCR) assays have gained increasing clinical use in the management of suspected severe pneumonia and other lower respiratory tract infections (9–11). These syndromic panels allow for the simultaneous detection of a broad spectrum of bacteria, respiratory viruses, and atypical pathogens within a matter of hours (12). Recent systematic reviews and meta-analyses have shown strong diagnostic utility of mPCR in suspected VAP and improvements in process-of-care endpoints in critically ill patients with pneumonia (6, 8). Specifically, the high negative predictive value of pneumonia mPCR panels can support antimicrobial stewardship by helping clinicians safely rule out bacterial infection and identify opportunities for diagnostic-driven de-escalation (13). However, interpreting these highly sensitive assays presents a distinct challenge. A positive nucleic acid signal does not universally equate to an active infection; rather, it requires careful contextualization against specimen quality and host immune status to differentiate true infection from colonization (14). More importantly, while the diagnostic yield of mPCR is well established, high-quality evidence evaluating whether the complexity of these pathogen detection profiles—such as the cumulative detection burden—provides prognostic information for hard clinical outcomes such as mortality remains limited (15).

Due to a combination of underlying chronic lung diseases, compromised immunity, invasive mechanical ventilation, and extensive prior antibiotic exposure, multi-pathogen co-detections are a frequent reality in critically ill patients (16). However, much of the existing literature is still organized around single pathogens or single pathogen classes, including studies that focus exclusively on respiratory viral co-infections (17). As a result, the clinical meaning of cross-category co-detection patterns involving bacteria, viruses, and other non-bacterial pathogens, as well as their potential prognostic relevance in adult ICU cohorts, remains insufficiently defined (18). In particular, it remains unclear whether a simple pathogen detection count from multiplex panels is associated with mortality risk after accounting for illness severity and key clinical exposures, even though emerging microbiome studies suggest that respiratory microbial complexity and dysbiosis are linked to disease severity and clinical outcomes in critically ill patients. In addition, because pathogen distribution in ICU pneumonia is shaped by local microbial ecology, care pathways, and shifting admission patterns, characterization of these co-detection structures in a real-world cohort may provide useful clinical and epidemiologic context.

Addressing these critical knowledge gaps, this retrospective cohort study was designed to characterize respiratory pathogen detection profiles and predominant co-detection patterns using rapid mPCR in ICU patients with suspected pulmonary TB or complex respiratory infections. We analyzed variations in pathogen detection patterns across baseline clinical features, including age, sex, comorbidities, and Acute Physiology and Chronic Health Evaluation II (APACHE-II) strata. Finally, we evaluated whether cumulative pathogen detection burden was associated with in-hospital mortality and 60 day overall survival after accounting for illness severity and key pre-sampling clinical exposures. We hypothesized that higher pathogen detection count would be associated with increased short-term mortality, while recognizing that molecular detection does not necessarily indicate active infection and may also reflect host status, prior antimicrobial exposure, or healthcare-associated microbial ecology.

## RESULTS

### Study population and baseline characteristics

**Fig 1:**
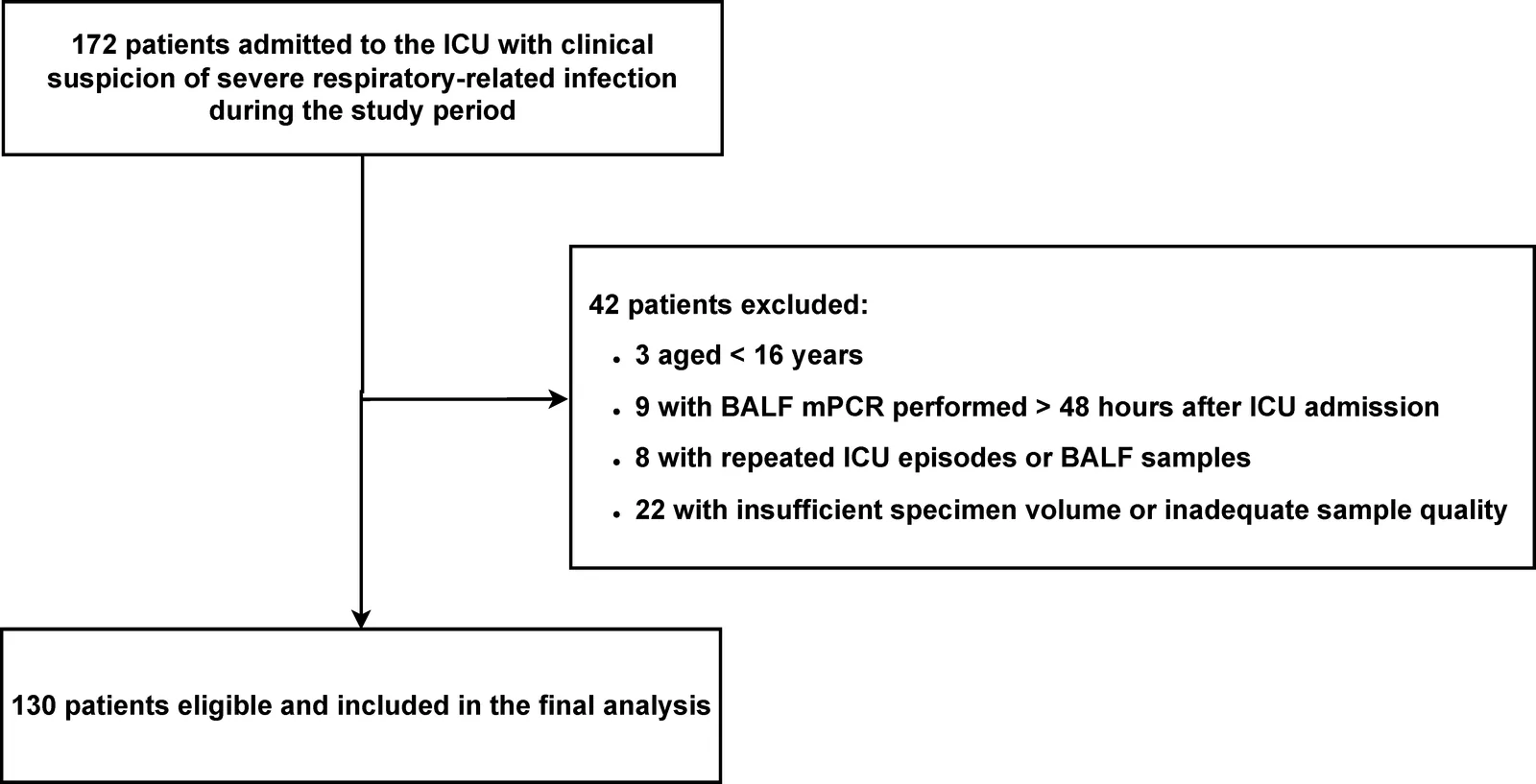
Flow diagram of the screening process.

A total of 172 patients were screened during the study period. We included 130 patients in the final analysis (Fig. 1). Forty-two patients were excluded from the cohort. Specifically, three patients were younger than 16 years. Nine patients had bronchoalveolar lavage fluid (BALF) multiplex PCR testing performed more than 48 h after intensive care unit admission. Eight patients provided repeated samples, and we retained only the first eligible sample for analysis. We excluded an additional 22 patients due to insufficient specimen volume or inadequate sample quality for testing.

The baseline demographic and clinical characteristics of the study cohort are summarized in detail (Table 1; Table S1). The mean age of the patients was 67.1 years with a standard deviation of 15.6 years. Patient ages ranged from 16 to 93 years. Males comprised 76.9% of the cohort, accounting for 100 out of the 130 patients. The mean Acute Physiology and Chronic Health Evaluation II score at ICU admission was 21.5 ± 8.3, consistent with a critically ill study population. The cohort exhibited several common comorbidities. Thirty-six patients had chronic obstructive pulmonary disease (COPD). Thirty-two patients had diabetes mellitus. Five patients had asthma. The mean intensive care unit length of stay was 20.6 days, with a range of 2 to 97 days. Twenty patients died during hospitalization, corresponding to an in-hospital mortality rate of 15.4%.

**TABLE 1:** Baseline demographic, clinical, treatment-exposure characteristics, and clinical outcomes of the study cohort

Characteristics	All patients (*n* = 130)	Min–Max
Age, mean ± SD, years	67.1 ± 15.6	16.0–93.0
Gender, *n*/*N* (%)		
Male	100/130 (76.9)	–^*a*^
Female	30/130 (23.1)	–
COPD, *n*/*N* (%)	36/128 (28.1)	–
Asthma, *n*/*N* (%)	5/128 (3.9)	–
Diabetes mellitus, *n*/*N* (%)	32/128 (25.0)	–
Immunocompromised status, *n*/*N* (%)	18/130 (13.8)	–
Prior anti-infective therapy within 7 days before BALF sampling, *n*/*N* (%)	65/130 (50.0)	–
Pre-ICU healthcare exposure, *n*/*N* (%)	89/130 (68.5)	–
Pre-ICU length of stay, mean ± SD, days	7.5 ± 9.9	0.0–60.0
APACHE-II score, mean ± SD	21.5 ± 8.3	4.0–46.0
ICU length of stay, mean ± SD, days	20.6 ± 18.1	2.0–97.0
In-hospital mortality, *n* (%)	20/130 (15.4)	–

^*a*^ “–” not applicable.

Overall multiplex PCR detection patterns revealed a high positivity rate (Table 2). The assay detected at least one pathogen category in 122 patients, representing a 93.8% overall detection rate. Eight patients tested negative for all evaluated targets. Bacteria constituted the most frequently detected category, present in 115 patients. The assay detected other pathogen targets in 55 patients and viral targets in 25 patients. We observed exclusive detections within a single pathogen category for a subset of patients. Fifty-six patients tested positive exclusively for bacteria. Five patients tested positive exclusively for other pathogens. No patients exhibited exclusive viral detection. Furthermore, 61 patients demonstrated mixed-category co-detection, accounting for 46.9% of the total cohort. Among these mixed detections, 11 patients had simultaneous bacterial and viral targets. Thirty-six patients had both bacteria and other pathogens. Two patients presented with viruses and other pathogens. Twelve patients exhibited triple-category co-detection, involving simultaneous positive signals for bacteria, viruses, and other pathogens. These findings highlight a remarkably high overall detection rate. They also reveal a substantial burden of mixed pathogen-category co-detections in bronchoalveolar lavage fluid samples from critically ill patients.

**TABLE 2:** Overall category-level pathogen detection patterns in BALF samples by multiplex PCR

Pathogen detection	*n* (%)
Bacterial targets	115 (88.5)
Viral targets	25 (19.2)
Other pathogen targets	55 (42.3)
At least one target detected	122 (93.8)
Only virus targets detected	0 (0.0)
Only bacteria targets detected	56 (43.1)
Only other targets detected	5 (3.8)
Any mixed-category positivity	61 (46.9)
Bacterial + viral co-detection	11 (8.5)
Bacterial + other co-detection	36 (27.7)
Viral + other co-detection	2 (1.5)
Triple-category positivity	12 (9.2)
Negative	8 (6.2)

The assay results demonstrated a high frequency of mixed-category co-detections. Consequently, we quantified the specific burden of target co-detection. We also mapped the detailed pathogen co-occurrence structures across all tested samples.

The bacterial target category included the bacterial pathogens listed in the assay panel (see Table 4 for detected bacterial targets in this cohort). The viral target category included respiratory viral targets listed in the assay panel (see Table 4 for detected viral targets in this cohort). The other pathogen targets category included: *Candida albicans* (Cal), *Legionella pneumophila* (Lpn), *Pneumocystis jirovecii* (Pji), *Chlamydia pneumoniae* (CP), and *Mycoplasma pneumoniae* (MP).

### Co-detection burden and pathogen co-occurrence patterns

The overall co-detection burden within the cohort is summarized in detail (Table 3). Among the 130 BALF samples, 26 (20.0%) showed single-target detection, 40 (30.8%) showed double-target detection, and 56 (43.1%) showed multiple-target detection (≥3 targets). These findings indicate that multi-pathogen detection was common in this ICU cohort, consistent with the high mixed-category positivity observed in the overall assay-level summary.

**TABLE 3:** Distribution of target detection burden per BALF sample by multiplex PCR

Detection burden per sample, *n* (%)	All patients (*n* = 130)
Negative detection	8 (6.2)
Single-target detection	26 (20.0)
Double-target detection	40 (30.8)
Multiple-target detection (≥3 targets)	56 (43.1)

**Fig 2:**
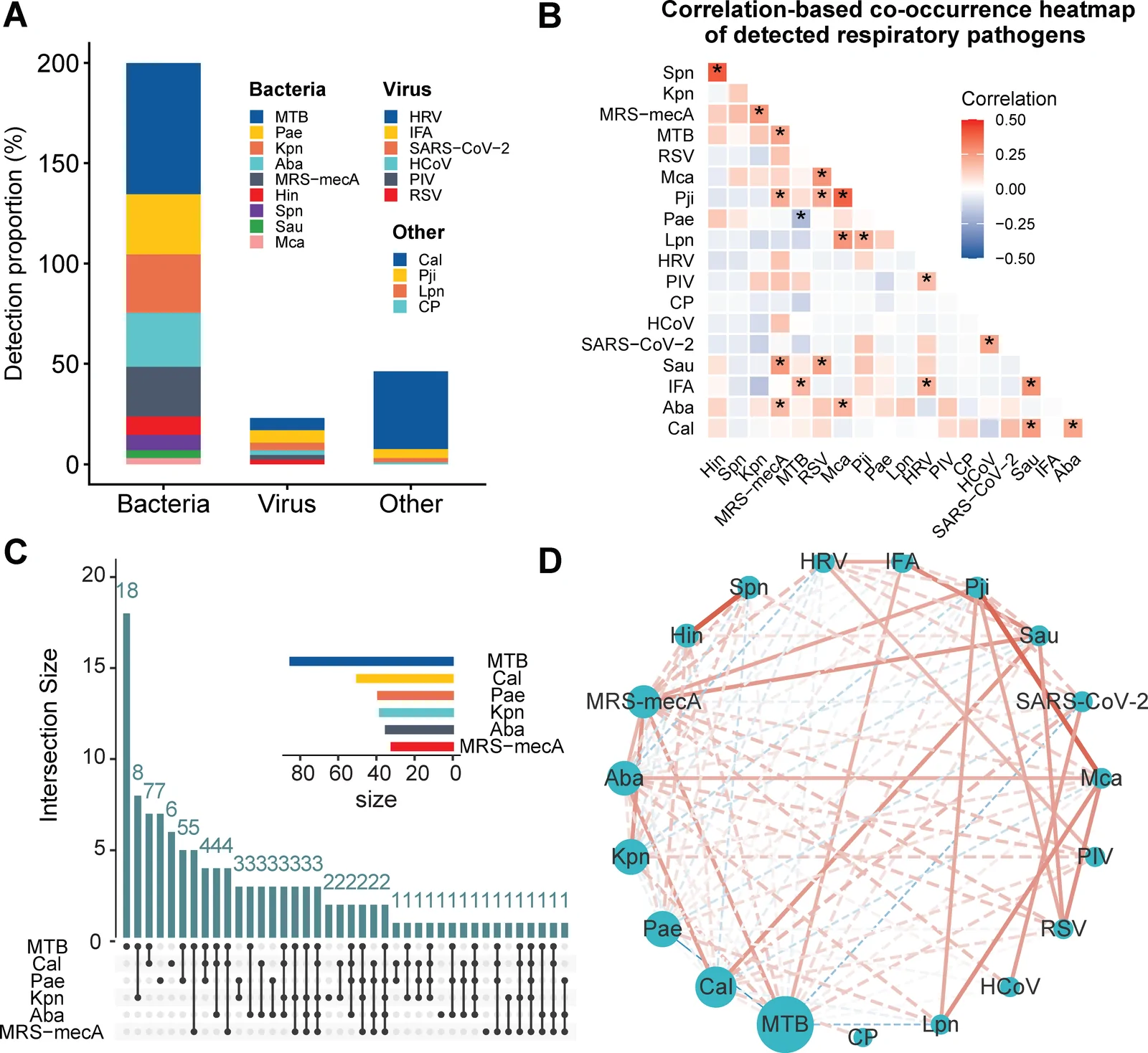
Co-detection burden and pathogen co-occurrence patterns in BALF samples. (**A**) Composition plot showing pathogen-specific detection frequencies grouped by category. Bar heights represent the summed proportions of pathogen detections within each category, and colored segments represent individual targets. (**B**) Correlation-based co-occurrence heatmap showing pairwise associations calculated from binary pathogen detection profiles across all BALF samples. Red indicates positive correlation, and blue indicates negative correlation. Asterisks indicate statistically significant correlations (*P* < 0.05). (**C**) UpSet plot illustrating target co-detection intersections. The plot is restricted to the six most frequently detected targets—MTB, Cal, Pae, Kpn, Aba, and MRS-*mecA*—to improve visual interpretability and reduce matrix sparsity. Intersections with zero counts were omitted. (**D**) Pathogen co-occurrence network summarizing pairwise correlations among detected pathogens. Red edges indicate positive correlations, and blue edges indicate negative correlations. Edge width is proportional to the absolute correlation coefficient. Solid lines represent statistically significant associations, whereas dashed lines represent non-significant associations. Node size reflects the number of samples in which the corresponding pathogen was detected. BALF, bronchoalveolar lavage fluid; HRV, human rhinovirus; IFA, influenza A virus; HCoV, human coronavirus; PIV, parainfluenza virus; RSV, respiratory syncytial virus; SARS-CoV-2, severe acute respiratory syndrome coronavirus 2; MTB, *Mycobacterium tuberculosis*; Pae, *Pseudomonas aeruginosa*; Kpn, *Klebsiella pneumoniae*; Aba, *Acinetobacter baumannii*; MRS-*mecA*, methicillin-resistant staphylococcal target with *mecA* marker; Hin, *Haemophilus influenzae*; Spn, Streptococcus pneumoniae; Sau, *Staphylococcus aureus*; Mca, *Moraxella catarrhalis*; Cal, *Candida albicans*; Pji, *Pneumocystis jirovecii*; Lpn, *Legionella pneumophila*; CP, *Chlamydia pneumoniae*.

The pathogen-specific detection spectrum is detailed across the cohort (Table 4) and visualized as a composition plot (Fig. 2A). Among bacterial targets, *Mycobacterium tuberculosis* (MTB) was the most frequently detected pathogen (85/130, 65.4%), followed by *Pseudomonas aeruginosa* (Pae) (30.0%), *Klebsiella pneumoniae* (Kpn) (29.2%), *Acinetobacter baumannii* (Aba) (26.9%), and methicillin-resistant staphylococcal target with *mecA* marker (MRS-*mecA*) (24.6%). Among viral targets, HRV and influenza A virus (IFA) were the most frequently detected (both 6.2%), followed by SARS-CoV-2 (3.8%); HCoV, PIV, and RSV were each detected in 2.3% of samples. Within the other pathogens category, Cal was the predominant target (50/130, 38.5%), whereas Pji (4.6%), Lpn (2.3%), and CP (0.8%) were less frequent. As shown in Table 4, the reported percentages include both single-pathogen detections and detections occurring within mixed-pathogen combinations.

**TABLE 4:** Pathogen-specific detection frequencies in BALF samples by multiplex PCR^*a*^

Pathogen detection	Detected samples, *n*	Proportion of all samples, %	Proportion of mPCR-positive samples, %
Bacterial targets			
MTB	85	65.4	69.7
Pae	39	30	32
Kpn	38	29.2	31.1
Aba	35	26.9	28.7
MRS-*mecA*	32	24.6	26.2
Hin	12	9.2	9.8
Spn	10	7.7	8.2
Sau	5	3.8	4.1
Mca	4	3.1	3.3
Viral targets			
HRV	8	6.2	6.6
IFA	8	6.2	6.6
SARS-CoV-2	5	3.8	4.1
HCoV	3	2.3	2.5
PIV	3	2.3	2.5
RSV	3	2.3	2.5
Other pathogen targets			
Cal	50	38.5	41
Pji	6	4.6	4.9
Lpn	3	2.3	2.5
CP	1	0.8	0.8

^*a*^ Percentages in the “Proportion of all samples” column were calculated using all 130 enrolled patients as the denominator. Percentages in the “Proportion of mPCR-positive samples” column were calculated using the 122 samples with at least one detected target as the denominator. Detection frequencies include targets detected as single findings or as part of mixed-target co-detection patterns. Pathogen targets are grouped and presented in the following order: bacterial targets, viral targets, and other pathogen targets.

Pairwise pathogen co-occurrence analysis identified 21 statistically significant pathogen pairs (Table 5; Fig. 2B). The correlation-based heatmap showed predominantly localized positive associations rather than large block-like pathogen clusters, indicating that co-detection patterns were mainly driven by specific pathogen pairs or small pathogen groups. The strongest positive correlations included Hin-Spn (*r* = 0.407), Mca-Pji (*r* = 0.385), and Sau-IFA (*r* = 0.282). Additional positive associations were also observed for several higher-frequency bacterial targets, including Kpn-MRS-*mecA* (*r* = 0.261), Aba-Cal (*r* = 0.233), and MRS-*mecA*-MTB (*r* = 0.228). In contrast, a significant negative correlation was observed for MTB-Pae (*r* = −0.194). The co-occurrence network provided a complementary summary of these pairwise associations (Fig. 2D).

**TABLE 5:** Statistically significant pairwise pathogen co-occurrence correlations ranked by correlation coefficient^*a*^

Pathogen A	Pathogen B	Co-detected samples, *n*	Correlation coefficient (*r*)	*P* value
Hin	Spn	5	0.407	<0.001
Mca	Pji	2	0.385	<0.001
Sau	IFA	2	0.282	0.001
Mca	RSV	1	0.269	0.002
Mca	Lpn	1	0.269	0.002
Kpn	MRS-*mecA*	16	0.261	0.003
MRS-*mecA*	Sau	4	0.257	0.003
Sau	Cal	5	0.253	0.004
Sau	RSV	1	0.236	0.007
HCoV	SARS-CoV-2	1	0.236	0.007
Aba	Cal	20	0.233	0.008
MRS-*mecA*	MTB	27	0.228	0.009
MRS-*mecA*	Pji	4	0.215	0.014
RSV	Pji	1	0.210	0.016
Lpn	Pji	1	0.210	0.016
HRV	IFA	2	0.201	0.022
MTB	Pae	20	−0.194	0.027
Aba	Mca	3	0.193	0.028
MTB	IFA	8	0.186	0.034
Aba	MRS-*mecA*	13	0.177	0.044
HRV	PIV	1	0.174	0.048

^*a*^ Pairwise correlations were calculated using binary presence data for each pathogen target across all BALF samples (*N* = 130). Only statistically significant pathogen pairs (*P* < 0.05) are shown and ranked in descending order of correlation coefficient. “Co-detected samples, *n*” indicates the number of samples in which both pathogen targets were detected. Pairs involving non-estimable correlations due to insufficient variability were excluded from the correlation analysis.

Higher-order co-detection patterns are detailed extensively in the supplementary materials. Triple-target combinations are provided in Table S2, while quadruple-target combinations are listed in Table S3. Among triple-target combinations, the most frequent patterns were Kpn + MRS-*mecA* + MTB (*n* = 15), Aba + MTB + Cal (*n* = 14), and Aba + Kpn + MTB (*n* = 12), followed by several MTB-centered combinations involving Cal, Pae, or MRS-*mecA*. Among quadruple-target combinations, the most common pattern was Aba + Kpn + MRS-*mecA* + MTB (*n* = 8), followed by Aba + Kpn + MTB + Cal (*n* = 7) and Kpn + MRS-*mecA* + MTB + Pae (*n* = 5). Because the full combinatorial matrix was highly sparse and many intersections had zero counts, the UpSet plot in Fig. 2C was restricted to the six most frequently detected targets (all detected in >30 samples) to improve interpretability.

Together, these results demonstrate a high burden of multi-target detection and recurrent co-detection patterns centered mainly on MTB, Aba, Kpn, MRS-*mecA*, and Cal in BALF samples from critically ill patients.

### Stratified epidemiologic features across demographic and clinical subgroups

**Fig 3:**
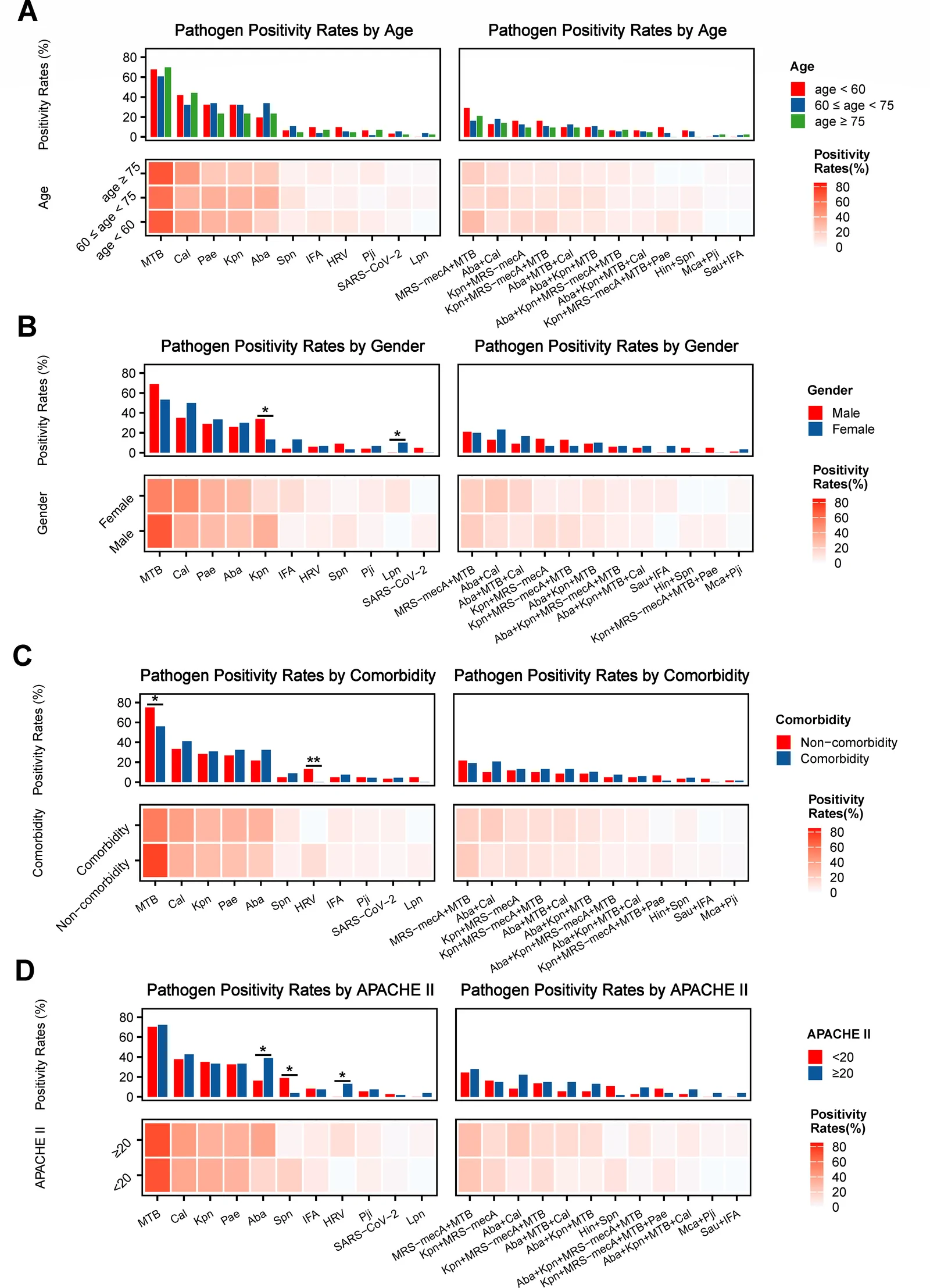
Stratified pathogen positivity profiles across demographic and clinical subgroups. (**A**) Age-stratified positivity rates across three age groups (<60 years, 60–75 years, and ≥75 years) for selected single pathogen targets and representative co-detection combinations. (**B**) Sex-stratified positivity rates (male vs female) for selected single targets and representative co-detection combinations. (**C**) Comorbidity-stratified positivity rates (non-comorbidity vs comorbidity) for selected single targets and representative co-detection combinations. (**D**) APACHE-II-stratified positivity rates (<20 vs ≥20) for selected single targets and representative co-detection combinations after completion of APACHE-II scores for all 130 patients. * indicates statistical significance (*P* < 0.05) for the corresponding comparison. Positivity rates are calculated as the proportion of patients within each subgroup with the indicated target or co-detection pattern. Heatmap color intensity represents positivity rate (%) within each subgroup. BALF, bronchoalveolar lavage fluid; ICU, intensive care unit; APACHE-II, Acute Physiology and Chronic Health Evaluation II; MTB, *Mycobacterium tuberculosis*; Kpn, *Klebsiella pneumoniae*; Aba, *Acinetobacter baumannii*; Spn, *Streptococcus pneumoniae*; HRV, human rhinovirus; Lpn, *Legionella pneumophila*; and other abbreviations as defined in Fig. 2.

Stratified analyses were performed to characterize pathogen detection patterns across age groups, sex, ICU length of stay, disease comorbidity status, COPD status, diabetes mellitus status, and APACHE-II strata (Tables 6 to 11, Fig. 3; Tables S4 to S7). Overall, most category-level detection indicators and pathogen-specific detection rates were broadly comparable across subgroups, with only a limited number of statistically significant differences.

**TABLE 6:** Age-stratified pathogen detection patterns and co-detection features in BALF samples

Pathogen detection	Age < 60, *n* (%) (*n* = 31)	60 ≤ Age < 75, *n* (%) (*n* = 56)	Age ≥ 75, *n* (%) (*n* = 43)	*P* value
Bacterial targets	26 (83.9)	51 (91.1)	38 (88.4)	0.602^*a*^
MTB	21 (67.7)	34 (60.7)	30 (69.8)	0.612^*a*^
Pae	10 (32.3)	19 (33.9)	10 (23.3)	0.492^*a*^
Kpn	10 (32.3)	18 (32.1)	10 (23.3)	0.574^*a*^
Aba	6 (19.4)	19 (33.9)	10 (23.3)	0.273^*a*^
MRS-*mecA*	9 (29.0)	12 (21.4)	11 (25.6)	0.721^*a*^
Hin	3 (9.7)	5 (8.9)	4 (9.3)	1.000
Spn	2 (6.5)	6 (10.7)	2 (4.7)	0.587
Sau	1 (3.2)	1 (1.8)	3 (7.0)	0.445
Mca	0 (0.0)	3 (5.4)	1 (2.3)	0.555
Viral targets	6 (19.4)	10 (17.9)	9 (20.9)	0.929^*a*^
HRV	3 (9.7)	3 (5.4)	2 (4.7)	0.728
IFA	3 (9.7)	2 (3.6)	3 (7.0)	0.475
SARS-CoV-2	1 (3.2)	3 (5.4)	1 (2.3)	0.859
HCoV	0 (0.0)	2 (3.6)	1 (2.3)	0.780
PIV	0 (0.0)	1 (1.8)	2 (4.7)	0.598
RSV	0 (0.0)	1 (1.8)	2 (4.7)	0.605
Other pathogen targets	14 (45.2)	20 (35.7)	21 (48.8)	0.396^*a*^
Cal	13 (41.9)	18 (32.1)	19 (44.2)	0.428^*a*^
Pji	2 (6.5)	1 (1.8)	3 (7.0)	0.381
Lpn	0 (0.0)	2 (3.6)	1 (2.3)	0.792
CP	1 (3.2)	0 (0.0)	0 (0.0)	0.257
At least one target detected	28 (90.3)	53 (94.6)	41 (95.3)	0.727
Only bacteria targets detected	11 (35.5)	28 (50.0)	17 (39.5)	0.360^*a*^
Only other targets detected	2 (6.5)	1 (1.8)	2 (4.7)	0.530
Any mixed-category positivity	15 (48.4)	24 (42.9)	22 (51.2)	0.702^*a*^
Bacterial + viral co-detection	3 (9.7)	5 (8.9)	3 (7.0)	0.924
Bacterial + other co-detection	9 (29.0)	14 (25.0)	13 (30.2)	0.831^*a*^
Viral + other co-detection	0 (0.0)	1 (1.8)	1 (2.3)	1.000
Triple-category positivity	3 (9.7)	4 (7.1)	5 (11.6)	0.742

^*a*^ *P* values were calculated using *χ*² test; all others were calculated using Fisher’s exact test.

**TABLE 7:** Sex-stratified pathogen detection patterns and co-detection features in BALF samples

Pathogen detection	Male, *n* (%) (*n* = 100)	Female, *n* (%) (*n* = 30)	*P* value
Bacterial targets	91 (91.0)	24 (80.0)	0.184^*a*^
MTB	69 (69.0)	16 (53.3)	0.173^*a*^
Pae	29 (29.0)	10 (33.3)	0.820^*a*^
Kpn	34 (34.0)	4 (13.3)	0.038
Aba	26 (26.0)	9 (30.0)	0.843^*a*^
MRS-*mecA*	25 (25.0)	7 (23.3)	1.000^*a*^
Hin	10 (10.0)	2 (6.7)	0.732
Spn	9 (9.0)	1 (3.3)	0.452
Sau	3 (3.0)	2 (6.7)	0.326
Mca	3 (3.0)	1 (3.3)	1.000
Viral targets	20 (20.0)	5 (16.7)	0.887^*a*^
HRV	6 (6.0)	2 (6.7)	1.000
IFA	4 (4.0)	4 (13.3)	0.082
SARS-CoV-2	5 (5.0)	0 (0.0)	0.589
HCoV	3 (3.0)	0 (0.0)	1.000
PIV	3 (3.0)	0 (0.0)	1.000
RSV	3 (3.0)	0 (0.0)	1.000
Other pathogen targets	38 (38.0)	17 (56.7)	0.109^*a*^
Cal	35 (35.0)	15 (50.0)	0.205^*a*^
Pji	4 (4.0)	2 (6.7)	0.621
Lpn	0 (0.0)	3 (10.0)	0.011
CP	0 (0.0)	1 (3.3)	0.231
At least one target detected	95 (95.0)	27 (90.0)	0.385
Only bacteria targets detected	48 (48.0)	8 (26.7)	0.063^*a*^
Only other targets detected	2 (2.0)	3 (10.0)	0.080
Any mixed-category positivity	45 (45.0)	16 (53.3)	0.553^*a*^
Bacterial + viral co-detection	9 (9.0)	2 (6.7)	1.000
Bacterial + other co-detection	25 (25.0)	11 (36.7)	0.308^*a*^
Viral + other co-detection	2 (2.0)	0 (0.0)	1.000
Triple-category positivity	9 (9.0)	3 (10.0)	1.000

^*a*^ *P* values were calculated using *χ*² test; all others were calculated using Fisher’s exact test.

**TABLE 8:** Pathogen detection patterns stratified by ICU length of stay (median cutoff: 15 days)

Pathogen detection	Short stay, *n* (%) (*n* = 70)	Long stay, *n* (%) (*n* = 60)	*P* value
Bacterial targets	61 (87.1)	54 (90.0)	0.816^*a*^
MTB	45 (64.3)	40 (66.7)	0.921^*a*^
Pae	21 (30.0)	18 (30.0)	1.000^*a*^
Kpn	21 (30.0)	17 (28.3)	0.988^*a*^
Aba	17 (24.3)	18 (30.0)	0.593^*a*^
MRS-*mecA*	15 (21.4)	17 (28.3)	0.480^*a*^
Hin	7 (10.0)	5 (8.3)	0.981^*a*^
Spn	6 (8.6)	4 (6.7)	0.752
Sau	3 (4.3)	2 (3.3)	1.000
Mca	3 (4.3)	1 (1.7)	0.624
Viral targets	18 (25.7)	7 (11.7)	0.071^*a*^
HRV	5 (7.1)	3 (5.0)	0.725
IFA	6 (8.6)	2 (3.3)	0.286
SARS-CoV-2	2 (2.9)	3 (5.0)	0.662
HCoV	3 (4.3)	0 (0.0)	0.249
PIV	3 (4.3)	0 (0.0)	0.249
RSV	2 (2.9)	1 (1.7)	1.000
Other pathogen targets	25 (35.7)	30 (50.0)	0.143^*a*^
Cal	21 (30.0)	29 (48.3)	0.050^*a*^
Pji	4 (5.7)	2 (3.3)	0.686
Lpn	2 (2.9)	1 (1.7)	1.000
CP	0 (0.0)	1 (1.7)	0.462
At least one target detected	65 (92.9)	57 (95.0)	0.725
Only bacteria targets detected	30 (42.9)	26 (43.3)	1.000^*a*^
Only other targets detected	3 (4.3)	2 (3.3)	1.000
Any mixed-category positivity	32 (45.7)	29 (48.3)	0.903^*a*^
Bacterial + viral co-detection	10 (14.3)	1 (1.7)	0.011
Bacterial + other co-detection	14 (20.0)	22 (36.7)	0.055^*a*^
Viral + other co-detection	1 (1.4)	1 (1.7)	1.000
Triple-category positivity	7 (10.0)	5 (8.3)	0.981^*a*^

^*a*^ *P* values were calculated using *χ*² test; all others were calculated using Fisher’s exact test.

**TABLE 9:** Pathogen detection patterns stratified by APACHE-II score (cutoff: 20) among patients with available APACHE-II data, *n* = 130

Pathogen detection	<20, *n* (%) (*n* = 59)	≥20, *n* (%) (*n* = 71)	*P* value
Bacterial targets	47 (79.7)	68 (95.8)	0.005
MTB	34 (57.6)	51 (71.8)	0.131^*a*^
Pae	17 (28.8)	22 (31.0)	0.939^*a*^
Kpn	16 (27.1)	22 (31.0)	0.773^*a*^
Aba	8 (13.6)	27 (38.0)	0.003^*a*^
MRS-*mecA*	11 (18.6)	21 (29.6)	0.216^*a*^
Hin	6 (10.2)	6 (8.5)	0.974^*a*^
Spn	8 (13.6)	2 (2.8)	0.042
Sau	0 (0.0)	5 (7.0)	0.063
Mca	1 (1.7)	3 (4.2)	0.626
Viral targets	6 (10.2)	19 (26.8)	0.030^*a*^
HRV	1 (1.7)	7 (9.9)	0.071
IFA	2 (3.4)	6 (8.5)	0.291
SARS-CoV-2	4 (6.8)	1 (1.4)	0.176
HCoV	1 (1.7)	2 (2.8)	1.000
PIV	0 (0.0)	3 (4.2)	0.251
RSV	0 (0.0)	3 (4.2)	0.251
Other pathogen targets	23 (39.0)	32 (45.1)	0.602^*a*^
Cal	22 (37.3)	28 (39.4)	0.944^*a*^
Pji	1 (1.7)	5 (7.0)	0.220
Lpn	1 (1.7)	2 (2.8)	1.000
CP	1 (1.7)	0 (0.0)	0.454
At least one target detected	54 (91.5)	68 (95.8)	0.467
Only bacteria targets detected	28 (47.5)	28 (39.4)	0.458^*a*^
Only other targets detected	5 (8.5)	0 (0.0)	0.017
Any mixed-category positivity	21 (35.6)	40 (56.3)	0.029^*a*^
Bacterial + viral co-detection	3 (5.1)	8 (11.3)	0.343
Bacterial + other co-detection	15 (25.4)	21 (29.6)	0.741^*a*^
Viral + other co-detection	2 (3.4)	0 (0.0)	0.204
Triple-category positivity	1 (1.7)	11 (15.5)	0.006

^*a*^ *P* values were calculated using *χ*² test; all others were calculated using Fisher’s exact test.

**TABLE 10:** COPD-stratified pathogen detection patterns and co-detection features, *n* = 128

Pathogen detection	Non-COPD, *n* (%) (*n* = 92)	COPD, *n* (%) (*n* = 36)	*P* value
Bacterial targets	84 (91.3)	29 (80.6)	0.163^*a*^
MTB	67 (72.8)	16 (44.4)	0.005^*a*^
Pae	25 (27.2)	13 (36.1)	0.435^*a*^
Kpn	30 (32.6)	8 (22.2)	0.347^*a*^
Aba	27 (29.3)	8 (22.2)	0.553^*a*^
MRS-*mecA*	25 (27.2)	6 (16.7)	0.309^*a*^
Hin	8 (8.7)	3 (8.3)	1.000
Spn	4 (4.3)	5 (13.9)	0.116
Sau	5 (5.4)	0 (0.0)	0.321
Mca	4 (4.3)	0 (0.0)	0.576
Viral targets	19 (20.7)	6 (16.7)	0.792^*a*^
HRV	8 (8.7)	0 (0.0)	0.104
IFA	6 (6.5)	2 (5.6)	1.000
SARS-CoV-2	4 (4.3)	1 (2.8)	1.000
HCoV	1 (1.1)	2 (5.6)	0.191
PIV	2 (2.2)	1 (2.8)	1.000
RSV	3 (3.3)	0 (0.0)	0.559
Other pathogen targets	40 (43.5)	13 (36.1)	0.575^*a*^
Cal	36 (39.1)	12 (33.3)	0.685^*a*^
Pji	5 (5.4)	1 (2.8)	1.000
Lpn	3 (3.3)	0 (0.0)	0.559
CP	0 (0.0)	1 (2.8)	0.281
At least one target detected	87 (94.6)	33 (91.7)	0.686
Only bacteria targets detected	39 (42.4)	17 (47.2)	0.766^*a*^
Only other targets detected	2 (2.2)	3 (8.3)	0.135
Any mixed-category positivity	46 (50.0)	13 (36.1)	0.222^*a*^
Bacterial + viral co-detection	8 (8.7)	3 (8.3)	1.000
Bacterial + other co-detection	27 (29.3)	7 (19.4)	0.359
Viral + other co-detection	1 (1.1)	1 (2.8)	0.485
Triple-category positivity	10 (10.9)	2 (5.6)	0.507

^*a*^ *P* values were calculated using *χ*² test; all others were calculated using Fisher’s exact test.

Age-stratified results are summarized in Table 6 (and visualized in Fig. 3A; selected plotting data in Table S4). Across the three age groups (<60 years, 60–75 years, and ≥75 years), there were no statistically significant differences in the detection rates of bacterial, viral, or other pathogen categories, nor in major single-pathogen targets including MTB, Pae, Kpn, Aba, HRV, IFA, SARS-CoV-2, and Cal (all *P* > 0.05). Similarly, no significant age-related differences were observed for major co-detection patterns, including selected pairwise, triple-target, and quadruple-target combinations shown in Table S4. These findings suggest that the overall pathogen spectrum and major co-detection structures were relatively stable across age strata in this ICU cohort.

Sex-stratified results are shown in Table 7 and Fig. 3B (selected plotting data in Table S5). Most pathogen-category indicators and individual pathogen detection rates did not differ significantly between males and females. However, Kpn was detected more frequently in males than in females (34.0% vs 13.3%, *P* = 0.038), whereas Lpn was detected only in females (10.0% vs 0.0%, *P* = 0.011). A borderline difference was also observed for the Sau + IFA pair in the plotting data set (0.0% vs 6.7%, *P* = 0.052, Table S5), although this did not meet the prespecified significance threshold. No significant sex-related differences were observed for the major high-frequency co-detection combinations.

Patients were stratified by ICU length of stay using the cohort median (15 days) as the cutoff (Table 8). Most category-level and pathogen-specific detection rates were similar between the short-stay and long-stay groups. Among category-level co-detection patterns, bacteria + virus co-detection was more frequent in the short-stay group than in the long-stay group (14.3% vs 1.7%, *P* = 0.011). At the pathogen level, Cal detection was more common in the long-stay group and reached borderline statistical significance (48.3% vs 30.0%, *P* = 0.050). No other significant differences were identified.

APACHE-II-stratified analyses were performed based on the baseline scores for all 130 patients, using a cutoff of 20 (Table 9; Fig. 3D; selected plotting data in Table S7). Compared with patients with APACHE-II scores <20, those with APACHE-II scores ≥20 had higher positivity rates for bacterial targets (95.8% vs 79.7%, *P* = 0.005) and viral targets (26.8% vs 10.2%, *P* = 0.030), whereas other pathogen target positivity did not differ significantly between groups (45.1% vs 39.0%, *P* = 0.602).

Several differences were observed at the co-detection and pathogen-specific levels. Any mixed-category positivity was more frequent in the APACHE-II ≥20 group than in the APACHE-II <20 group (56.3% vs 35.6%, *P* = 0.029), as was triple-category positivity (15.5% vs 1.7%, *P* = 0.006). At the individual target level, Aba was more frequently detected in patients with APACHE-II ≥20 (38.0% vs 13.6%, *P* = 0.003), whereas Spn was less frequently detected in this group (2.8% vs 13.6%, *P* = 0.042). Only-other-target positivity was observed only in the APACHE-II <20 group (8.5% vs 0.0%, *P* = 0.017). These findings indicate that patients with higher APACHE-II scores showed more frequent mixed-category and triple-category detection patterns, supporting the need to adjust for illness severity in the exploratory risk-stratification analyses. Stratified analyses by comorbidity status are summarized in Fig. 3C and Table S7, while disease-specific subgroup analyses for COPD and diabetes mellitus are shown in Tables 10 and 11, respectively. In the COPD-stratified analysis (available data: *n* = 128), MTB detection was significantly less frequent in patients with COPD than in those without COPD (44.4% vs 72.8%, *P* = 0.005), whereas no other category-level indicators, individual pathogens, or co-detection patterns differed significantly. In the diabetes mellitus–stratified analysis (available data: *n* = 128), no statistically significant differences were identified across pathogen-category indicators, individual pathogens, or co-detection patterns (all *P* > 0.05).

**TABLE 11:** Diabetes mellitus-stratified pathogen detection patterns and co-detection features, *n* = 128

Pathogen detection	Non-diabetes, *n* (%) (*n* = 96)	Diabetes, *n* (%) (*n* = 32)	*P* value
Bacterial targets	83 (86.5)	30 (93.8)	0.354
MTB	60 (62.5)	23 (71.9)	0.454
Pae	30 (31.2)	8 (25.0)	0.655^*a*^
Kpn	24 (25.0)	14 (43.8)	0.074^*a*^
Aba	22 (22.9)	13 (40.6)	0.086^*a*^
MRS-*mecA*	21 (21.9)	10 (31.2)	0.404
Hin	8 (8.3)	3 (9.4)	1.000
Spn	6 (6.2)	3 (9.4)	0.690
Sau	4 (4.2)	1 (3.1)	1.000
Mca	2 (2.1)	2 (6.2)	0.260
Viral targets	17 (17.7)	8 (25.0)	0.520^*a*^
HRV	8 (8.3)	0 (0.0)	0.200
IFA	5 (5.2)	3 (9.4)	0.412
SARS-CoV-2	3 (3.1)	2 (6.2)	0.598
HCoV	3 (3.1)	0 (0.0)	0.573
PIV	2 (2.1)	1 (3.1)	1.000
RSV	1 (1.0)	2 (6.2)	0.154
Other pathogen targets	38 (39.6)	15 (46.9)	0.604^*a*^
Cal	34 (35.4)	14 (43.8)	0.527^*a*^
Pji	4 (4.2)	2 (6.2)	0.639
Lpn	3 (3.1)	0 (0.0)	0.573
CP	1 (1.0)	0 (0.0)	1.000
At least one target detected	90 (93.8)	30 (93.8)	1.000
Only bacteria targets detected	44 (45.8)	12 (37.5)	0.537^*a*^
Only other targets detected	5 (5.2)	0 (0.0)	0.330
Any mixed-category positivity	41 (42.7)	18 (56.2)	0.260^*a*^
Bacterial + viral co-detection	8 (8.3)	3 (9.4)	1.000
Bacterial + other co-detection	24 (25.0)	10 (31.2)	0.644^*a*^
Viral + other co-detection	2 (2.1)	0 (0.0)	1.000
Triple-category positivity	7 (7.3)	5 (15.6)	0.294^*a*^

^*a*^ *P* values were calculated using *χ*² test; all others were calculated using Fisher’s exact test.

In the broader comorbidity-group visualization data set (Table S7), MTB detection remained significantly different between non-comorbidity and comorbidity groups (75.0% vs 55.9%, *P* = 0.038). In addition, HRV detection was observed only in the non-comorbidity group (13.3% vs 0.0%, *P* = 0.002). Overall, subgroup differences were limited and were primarily driven by selected bacterial and viral targets rather than by global positivity metrics.

### Associations between pathogen detection features and clinical outcomes

To evaluate the clinical relevance of pathogen detection features, we analyzed associations with the primary endpoint, in-hospital mortality, using univariate comparisons, multivariable logistic regression, and time-to-event analyses.

Baseline clinical and exposure variables stratified by survival status are summarized in Table 12. Compared with survivors, patients who died in hospital were significantly older (74.2 ± 11.0 vs 65.8 ± 15.9 years, *P* = 0.006), had a significantly higher APACHE-II score (28.2 ± 6.6 vs 20.2 ± 8.0, *P* < 0.001), and had a shorter ICU length of stay (14.0 ± 8.7 vs 21.8 ± 19.1 days, *P* = 0.005). No significant differences were observed in sex, COPD, asthma, diabetes mellitus, immunocompromised status, prior anti-infective therapy within 7 days before BALF sampling, pre-ICU healthcare exposure, or pre-ICU hospitalization days between survivors and non-survivors.

**TABLE 12:** Baseline clinical characteristics stratified by in-hospital mortality

Variables	Survived	Death	Total	*P* value
Age, mean ± SD, years	65.8 ± 15.9	74.2 ± 11	67.1 ± 15.6	0.006
Gender, *n*/*N* (%)				0.610
Male	86/110 (78.2)	14/20 (70.0)	100/130 (76.9)	
Female	24/110 (21.8)	6/20 (30.0)	30/130 (23.1)	
COPD, *n*/*N* (%)	32/108 (29.6)	4/20 (20.0)	36/128 (28.1)	0.433
Asthma, *n*/*N* (%)	4/108 (3.7)	1/20 (5.0)	5/128 (3.9)	0.579
Diabetes mellitus, n/N (%)	25/108 (23.1)	7/20 (35.0)	32/128 (25.0)	0.399
Immunocompromised status, *n*/*N* (%)	15/110 (13.6)	3/20 (15)	18/130 (13.8)	1.000
Prior anti-infective therapy within 7 days, *n*/*N* (%)	53/110 (48.2)	12/20 (60.0)	65/130 (50.0)	0.466
Pre-ICU healthcare exposure, *n*/*N* (%)	76/110 (69.1)	13/20 (65.0)	89/130 (68.5)	0.920
Pre-ICU hospitalization days, mean ± SD, days	7.4 ± 10.1	8.1 ± 9.4	7.5 ± 9.9	0.755
APACHE-II score, mean ± SD	20.2 ± 8.0	28.2 ± 6.6	21.5 ± 8.3	<0.001
ICU length of stay, mean ± SD, days	21.8 ± 19.1	14 ± 8.7	20.6 ± 18.1	0.005

Associations between pathogen detection variables and in-hospital mortality are shown in Table 13. At the category level, viral target positivity was significantly more frequent among non-survivors than survivors (45.0% vs 14.5%, *P* = 0.004), and other pathogen target positivity was also more common in non-survivors (65.0% vs 38.2%, *P* = 0.047). Bacterial target positivity did not differ significantly between groups (100.0% vs 86.4%, *P* = 0.125).

**TABLE 13:** Pathogen detection features stratified by in-hospital mortality

Pathogen detection	Survived (*n* = 110)	Death (*n* = 20)	*P* value
Bacterial targets	95 (86.4)	20 (100.0)	0.125
Aba	26 (23.6)	9 (45.0)	0.088^*a*^
Hin	9 (8.2)	3 (15.0)	0.395
Kpn	32 (29.1)	6 (30.0)	1.000^*a*^
Mca	2 (1.8)	2 (10.0)	0.112
MRS-*mecA*	25 (22.7)	7 (35.0)	0.374^*a*^
MTB	72 (65.5)	13 (65.0)	1.000^*a*^
Pae	30 (27.3)	9 (45.0)	0.185^*a*^
Sau	2 (1.8)	3 (15.0)	0.026
Spn	9 (8.2)	1 (5.0)	1.000
Viral targets	16 (14.5)	9 (45.0)	0.004^*a*^
HCoV	3 (2.7)	0 (0.0)	1.000
HRV	4 (3.6)	4 (20.0)	0.019
IFA	5 (4.5)	3 (15.0)	0.105
PIV	2 (1.8)	1 (5.0)	0.397
RSV	1 (0.9)	2 (10.0)	0.062
SARS-CoV-2	5 (4.5)	0 (0.0)	1.000
Other pathogen targets	42 (38.2)	13 (65.0)	0.047^*a*^
Cal	39 (35.5)	11 (55.0)	0.161^*a*^
CP	1 (0.9)	0 (0.0)	1.000
Lpn	2 (1.8)	1 (5.0)	0.397
Pji	3 (2.7)	3 (15.0)	0.046

^*a*^ *P* values were calculated using *χ*² test; all others were calculated using Fisher’s exact test.

At the individual pathogen level, Sau (15.0% vs 1.8%, *P* = 0.026), HRV (20.0% vs 3.6%, *P* = 0.019), and Pji (15.0% vs 2.7%, *P* = 0.046) were significantly more frequently detected among non-survivors. Several additional targets showed non-significant trends, including Aba (45.0% vs 23.6%, *P* = 0.088) and RSV (10.0% vs 0.9%, *P* = 0.062).

**Fig 4:**
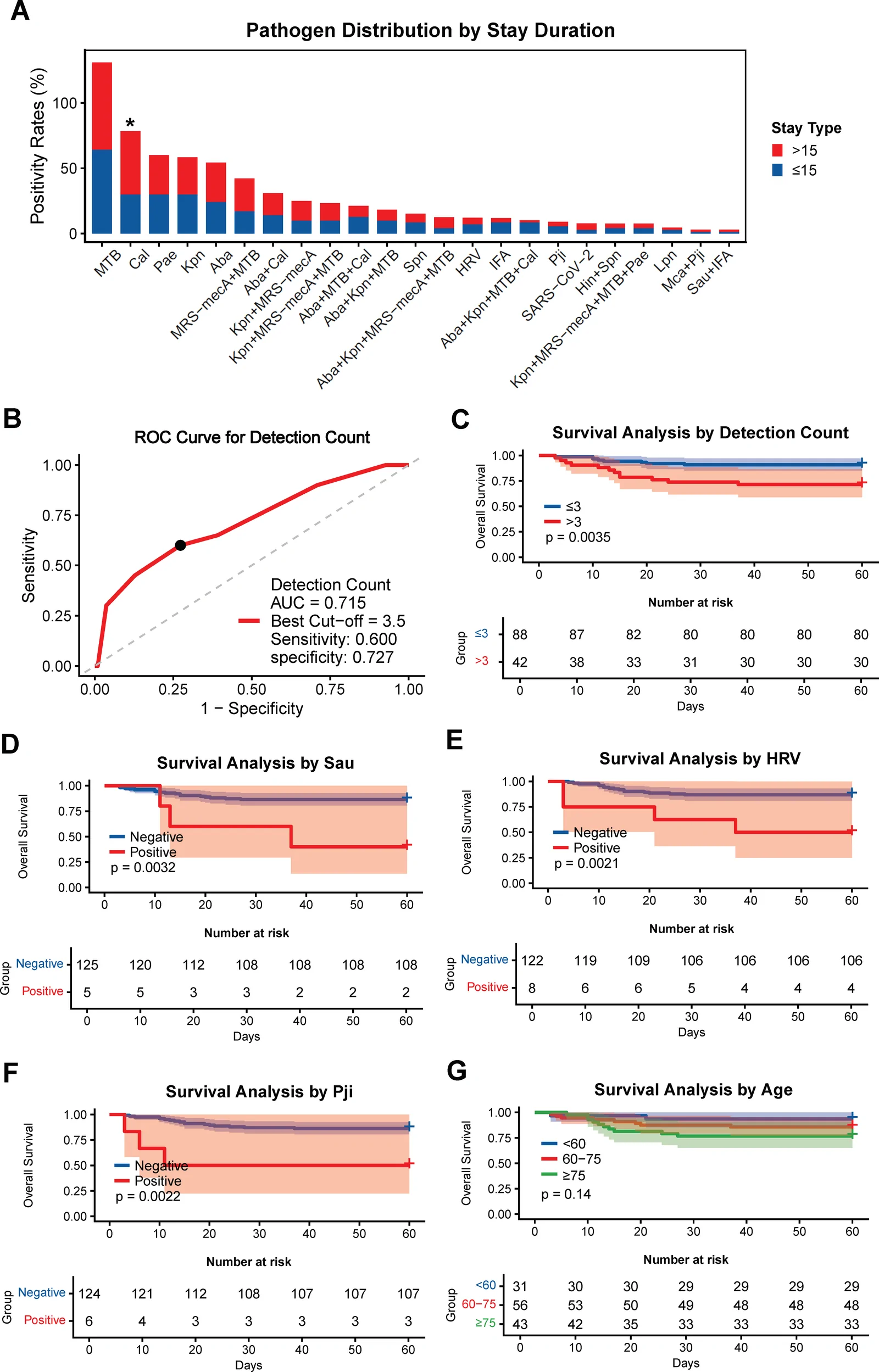
Pathogen detection burden, selected pathogen features, and survival outcomes in critically ill patients. (**A**) Pathogen distribution by ICU stay duration (short stay vs long stay; median cutoff = 15). Bars represent subgroup-specific positivity rates (%) for selected single pathogens and recurrent co-detection combinations. (**B**) ROC curve analysis for total pathogen detection count in predicting mortality. The Youden-derived threshold was 3.5, corresponding to a grouping of >3 vs ≤3 detected targets (AUC = 0.715). (**C**) Kaplan-Meier survival curves stratified by the ROC-derived detection-count threshold (≤3 vs >3 detected targets). (**D–F**) Kaplan-Meier survival curves stratified by positivity of Sau (**D**), HRV (**E**), and Pji (**F**). (**G**) Kaplan-Meier survival curves stratified by age group (<60 years, 60–75 years, and ≥75 years). For Kaplan-Meier panels, shaded areas indicate 95% confidence intervals, and *P* values are from log-rank tests. Numbers at risk are shown below each plot. ICU, intensive care unit; ROC, receiver operating characteristic; AUC, area under the curve; HRV, human rhinovirus; Pji, *Pneumocystis jirovecii*; Sau, *Staphylococcus aureus*; and other pathogen abbreviations are as defined in Fig. 2.

Time-to-event analyses over the 60 day follow-up period are shown in Fig. 4 and Fig. S1. Because the original Kaplan-Meier curves suggested a threshold-like relationship between pathogen detection burden and survival, we performed receiver operating characteristic (ROC) analysis to identify an exploratory threshold for total pathogen detection count. The Youden-derived threshold was 3.5, corresponding to a grouping of >3 vs ≤3 detected targets, with an AUC of 0.715 (95% CI, 0.586–0.844), sensitivity of 60.0%, and specificity of 72.7% (Fig. 4B). Using this ROC-derived threshold, patients with >3 detected targets had significantly poorer 60 day overall survival than those with ≤3 detected targets (Fig. 4C; log-rank *P* = 0.0035). The additional exploratory Kaplan-Meier curves using other empirical thresholds are provided in Fig. S1.

Positive detection of Sau, HRV, and Pji was each associated with significantly worse survival (Fig. 4D through F; log-rank *P* = 0.0032, 0.0021, and 0.0022, respectively). Survival did not differ significantly across age strata in the Kaplan-Meier analysis (Fig. 4G; log-rank *P* = 0.14). In addition, viral target positivity and other pathogen target positivity were evaluated in Fig. S1 and showed poorer survival in exploratory analyses.

As an exploratory stratified comparison by ICU stay duration, the distribution plot in Fig. 4A (see also Table S8) showed broadly similar pathogen profiles between short-stay and long-stay groups, with Cal reaching borderline significance (48.3% vs 30.0%, *P* = 0.050).

**Fig 5:**
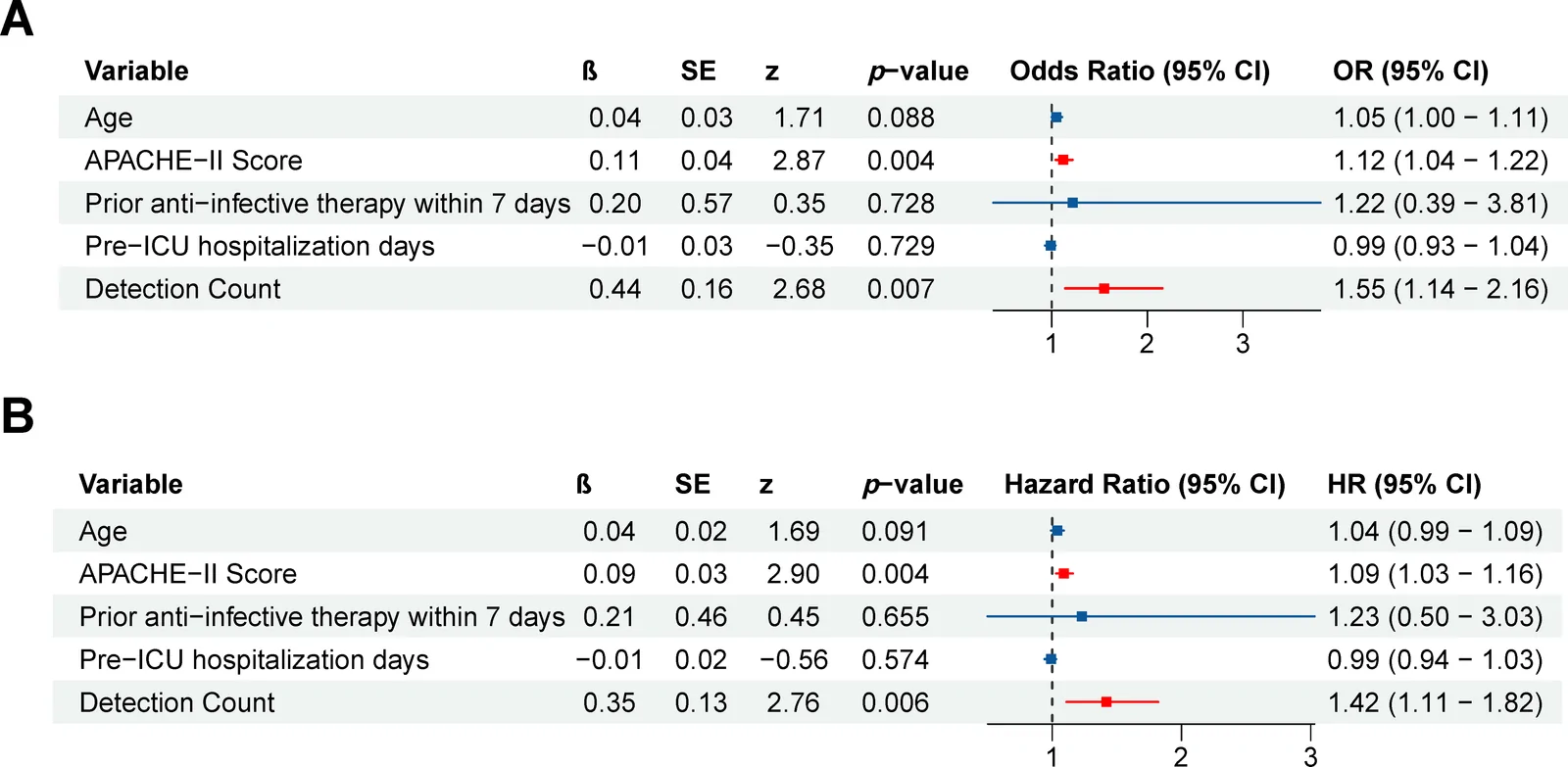
Multivariable regression analyses of factors associated with in-hospital mortality. (**A**) Forest plot of adjusted odds ratios (ORs) and 95% confidence intervals from the multivariable logistic regression model for in-hospital mortality. The model included age, APACHE-II score, prior anti-infective therapy within 7 days before BALF sampling, pre-ICU hospitalization days, and total pathogen detection count. (**B**) Forest plot of adjusted hazard ratios (HRs) and 95% confidence intervals from the multivariable Cox proportional hazards model on 60 day overall survival. The model included the same covariates. Prior anti-infective therapy was defined as documented systemic antibacterial, anti-tuberculosis, antifungal, or antiviral treatment within 7 days before BALF sampling. Pre-ICU hospitalization days were modeled as a continuous variable. Squares represent adjusted ORs or HRs, horizontal lines represent 95% confidence intervals, and the vertical dashed line indicates a ratio of 1.0.

We used multivariable regression models to evaluate whether total pathogen detection count remained associated with mortality after adjustment for clinical severity and key pre-sampling exposures (Fig. 5). In the logistic regression model for in-hospital mortality, total pathogen detection count was associated with increased odds of death (adjusted OR 1.55, 95% CI 1.14–2.16, *P* = 0.007). APACHE-II score was also significantly associated with in-hospital mortality, whereas prior anti-infective therapy and pre-ICU hospitalization days were not statistically significant in the adjusted model.

To evaluate time-to-event risk over the 60 day follow-up, we constructed a multivariable Cox proportional hazards model (Fig. 5B). ICU length of stay was not included in this model to avoid time-dependent bias. In the Cox model, total pathogen detection count remained associated with increased mortality hazard (adjusted HR 1.42, 95% CI 1.11–1.82, *P* = 0.006). These dual modeling approaches consistently support the prognostic association of the cumulative pathogen detection burden. Sensitivity analyses using alternative pathogen detection burden definitions further supported the stability of this association (Table S9). When the pathogen detection count was recalculated after excluding Cal, the association with outcomes remained statistically significant in the main adjusted model (adjusted OR 1.57, 95% CI 1.12–2.24, *P* = 0.010; adjusted HR 1.48, 95% CI 1.13–1.94, *P* = 0.005). Similar results were observed in models additionally adjusting for immunocompromised status and in analyses excluding immunocompromised patients. In an additional cycle threshold (Ct)-based sensitivity analysis, excluding detections with raw Ct values >35 from the pathogen count did not materially change the association (adjusted OR 1.48, 95% CI 1.09–2.06, *P* = 0.015; adjusted HR 1.41, 95% CI 1.09–1.82, *P* = 0.009). Available Ct values and internal-control-adjusted 2^−ΔCt^ signals are summarized in Table S10 as exploratory semi-quantitative context.

## DISCUSSION

In this retrospective cohort study of critically ill patients with severe lower respiratory tract symptoms and a high clinical suspicion of tuberculosis or complex pathogen co-occurrences, we utilized rapid mPCR to comprehensively map the respiratory pathogen landscape. Our principal findings reveal a remarkably high overall pathogen positivity rate (93.8%) and a substantial burden of mixed-category co-detections (46.9%). More importantly, cumulative pathogen detection burden—quantified as the total number of distinct targets identified by mPCR—remained associated with in-hospital mortality and 60 day overall survival after adjustment for age, APACHE-II score, prior anti-infective therapy, and pre-ICU hospitalization duration. Sensitivity analyses excluding *Candida albicans*, excluding immunocompromised patients, and excluding detections with raw Ct values >35 showed directionally consistent findings, supporting the stability of this association while underscoring its observational nature.

The microbial ecology observed in our cohort should be interpreted in the context of a clinically selected, TB-enriched ICU population rather than as representative of unselected ICU pneumonia populations (19). Consequently, our results may not be directly applicable to lower-risk pneumonia cohorts, non-TB-enriched clinical settings, or centers with fundamentally different antimicrobial ecologies. The exceptionally high detection rate of the *Mycobacterium tuberculosis* (65.4%) in our cohort is therefore best interpreted in light of local testing indications, where BALF mPCR was preferentially applied to critically ill patients with clinical or radiologic suspicion for TB or with poor response to initial empiric therapy. In addition, a TB-enriched cohort is biologically plausible for complex co-detection patterns. Post-TB structural lung damage and persistent immune dysregulation can create a permissive niche for recurrent bacterial and fungal detection and secondary infections (20). This interpretation is consistent with our co-occurrence results showing frequent MTB co-detections with opportunistic and nosocomial targets, particularly Aba, Kpn, and Cal. The prominent detection of Cal (38.5%) and its borderline association with longer ICU stay may reflect colonization pressure, prior anti-infective exposure, and critical illness-associated dysbiosis rather than invasive fungal disease in all cases (21, 22). Recent research supports that Cal airway colonization is common, increases over ICU stay, and is associated with worse prognosis and higher co-detection rates of Pae and Aba, while true *Candida* pneumonia remains uncommon and should not be presumed from respiratory detection alone (23–25).

While conventional clinical reasoning often seeks a single “culprit” pathogen, our survival analyses suggest that cumulative pathogen detection burden may provide exploratory risk-stratification information in this ICU population. Therefore, this metric should be interpreted as a risk-stratification signal rather than as evidence that polymicrobial infection *per se* directly causes mortality. Although causal inference is not possible in this retrospective study, several mechanisms may explain this pattern. First, polymicrobial co-detections—particularly cross-category detections—may reflect biologically relevant pathogen-pathogen and host-pathogen interactions that may amplify lung injury severity, including epithelial barrier disruption, impaired mucociliary clearance, and dysregulated inflammatory responses (26, 27). Second, a high mPCR target count may serve as a surrogate marker for profound host immune exhaustion, compromised airway clearance, or severe microbiome dysbiosis, conditions that intrinsically portend a poor prognosis in the ICU (19). Third, mixed detection of resistant or healthcare-associated organisms and less routinely covered targets may indicate a higher risk of initial therapeutic mismatch or delayed optimization; notably, recent randomized-trial meta-analysis data show that syndromic PCR improves adequacy and speed of effective antimicrobial therapy in critically ill pneumonia, even though hard outcome benefits such as mortality reduction remain inconsistent at the trial level (6, 15). Interestingly, several individual targets (notably *Staphylococcus aureus*, HRV, and *Pneumocystis jirovecii* in our cohort-level analyses) were also associated with worse survival. These pathogen-specific findings should be considered exploratory because sample sizes for several individual targets were small, and molecular detection alone does not establish active infection. This is directionally consistent with the growing recognition that HRV can cause severe lower respiratory disease in adults, including ICU-level illness, and that PJP in critically ill patients carries high short-term mortality (27). At the same time, the prognostic impact of respiratory viral detection in ventilated/nosocomial pneumonia is heterogeneous across cohorts, and some studies have not observed independent mortality effects for viral co-detection in bacterial VAP/HAP populations (6). Therefore, the clinical significance of viral positivity likely depends on host factors, timing, specimen context, and the broader microbial/ecological background.

Several limitations of our study must be acknowledged. First, this was a single-center retrospective study of a clinically selected, TB-enriched ICU cohort. Therefore, the pathogen distribution, co-detection structure, and prognostic associations should not be generalized directly to all ICU pneumonia populations, lower-risk pneumonia cohorts, non-TB-enriched settings, or centers with different antimicrobial ecology and testing practices. Second, and most importantly, the inherent hypersensitivity of mPCR assays presents a clinical challenge in distinguishing between active, tissue-invasive disease and airway colonization or nucleic acid shedding. We strictly utilized the term “detection” rather than “infection” throughout this study, particularly relevant for the high positivity rates of *Candida albicans* and certain respiratory viruses. Although available Ct values and internal-control-adjusted 2^-ΔCt^ values provided exploratory semi-quantitative context, these values should not be interpreted as standardized pathogen-load measurements because the assay was not designed as a quantitative pathogen-load platform, and target assignment depends on both amplification and melting-curve characteristics. Accordingly, Ct-based results were treated as exploratory, and the primary analyses remained based on qualitative detection count. Third, due to the retrospective design, we lacked granular longitudinal data on how mPCR results directly prompted subsequent antimicrobial de-escalation or escalation, which limits our ability to evaluate the interventional impact of the assay on survival trajectories. Fourth, although the multivariate models adjusted for APACHE-II score, prior anti-infective therapy, pre-ICU hospitalization duration, and immunocompromised status in sensitivity analyses, residual confounding remains possible. No clinically valid instrumental variable was available that would influence pathogen detection burden without also affecting mortality or illness severity. Thus, the observed association should not be interpreted as evidence that reducing pathogen detection burden alone would improve outcomes.

## MATERIALS AND METHODS

### Study design and population

This retrospective observational cohort study was conducted in the adult medical and respiratory intensive care units of Wuhan Pulmonary Hospital between July 2023 and January 2026. We screened adult patients aged 16 years and older who presented with severe lower respiratory tract symptoms. To be included, patients must have undergone bronchoscopy with bronchoalveolar lavage as part of routine clinical care within 48 h of intensive care unit admission. Furthermore, inclusion required a high clinical, epidemiological, or radiological suspicion of pulmonary tuberculosis, atypical pathogens, or complex pathogen co-occurrences failing to respond to initial empirical therapy. We excluded patients admitted to non-intensive care settings or pediatric units. Additional exclusion criteria involved insufficient residual fluid volume, invalid assay results, testing performed beyond the 48 h admission window, and missing key follow-up outcome data. For patients with repeated admissions or multiple samples, we retained only the first eligible intensive care episode and specimen for analysis.

### Setting and specimen collection

BALF specimens were systematically collected upon clinical suspicion of severe respiratory disease, with a median collection time occurring within 24 h of intensive care unit admission. In adherence to the Surviving Sepsis Campaign guidelines, clinicians obtained appropriate routine microbiologic cultures and molecular tests prior to initiating new antimicrobial therapy, strictly provided that this process did not substantially delay life-saving interventions. Residual fluid aliquots remaining after routine clinician-ordered diagnostic testing were subsequently utilized for the multiplex PCR assay. No additional bronchoscopic procedures were performed exclusively for research purposes. Data regarding anti-infective therapy before BALF sampling, including antibacterial, anti-tuberculosis, antifungal, and antiviral therapy, were retrospectively extracted from electronic medical records.

### Multiplex PCR assay validation

We analyzed the BALF using a single-tube multiplex fluorescent PCR coupled with melt-curve analysis based on MPA technology utilizing a Respiratory Panel (Biotron, Guangzhou, China). The system employed four channels comprising FAM, VIC, and ROX for specific targets alongside CY5 for the endogenous internal control. Each 20 µL reaction contained 15 µL of master mix and 5 µL of nucleic acid extract. Cycling and melting procedures followed the manufacturer’s instructions and were performed on a SLAN-96 real-time PCR system. Target positivity required a typical amplification curve with a cycle threshold of 38 or less, accompanied by a melt peak within the target-specific temperature range. Sample validation required the CY5 internal control to amplify at a cycle threshold of 38 or less. The comprehensive respiratory panel detected a broad spectrum of targets. Bacterial targets included the *Mycobacterium tuberculosis*, *Pseudomonas aeruginosa*, *Klebsiella pneumoniae*, *Acinetobacter baumannii*, the methicillin-resistant staphylococcal target carrying the *mecA* marker, *Haemophilus influenzae*, *Streptococcus pneumoniae*, *Staphylococcus aureus*, and *Moraxella catarrhalis*. Viral targets encompassed human rhinovirus, influenza A virus, severe acute respiratory syndrome coronavirus 2, human coronavirus, parainfluenza virus, and respiratory syncytial virus. Other pathogen targets included *Candida albicans*, *Pneumocystis jirovecii*, *Legionella pneumophila*, *Chlamydia pneumoniae*, and *Mycoplasma pneumoniae*. The microbiological readout was generated via standardized automated platforms, ensuring blinding from the subjective clinical assessments of the treating physicians. To explore available semi-quantitative information, raw Ct values were retrieved for positive detections with target-assignable amplification signals and categorized into pre-specified intervals (≤30, >30 to ≤35, and >35 to ≤38). Where corresponding internal-control Ct values were available, ΔCt was calculated as pathogen Ct minus internal-control Ct, and 2^−ΔCt^ was summarized as an exploratory internal-control-adjusted relative signal. Because this was a single-tube multiplex PCR assay coupled with melt-curve analysis, Ct and 2^−ΔCt^ values were considered exploratory semi-quantitative indicators rather than standardized quantitative pathogen-load measurements. For shared-channel or otherwise non-discretizable amplification signals, Ct-based metrics were not assigned to individual targets.

### Epidemiological and clinical variables

All baseline demographic, clinical, and laboratory data were extracted retrospectively from the hospital electronic health information system. The primary outcome was defined as in-hospital mortality, complemented by time-to-event overall survival analysis over a 60 day follow-up period. The primary exposure of interest was the multiplex PCR pathogen profile and the total pathogen detection count. We defined this count as the continuous sum of distinct pathogen targets identified within a single patient specimen. We categorized detected targets into bacteria, viruses, and other pathogens, with *Candida albicans*, *Legionella pneumophila*, and *Pneumocystis jirovecii* under the other pathogens category.

To adjust for potential confounding, we extracted baseline covariates including age, sex, specific comorbidities such as chronic obstructive pulmonary disease, asthma, and diabetes mellitus, and the APACHE-II score at ICU admission. APACHE-II scores were calculated for all 130 patients based on a comprehensive review of nursing records, laboratory results, arterial blood gas data, and vital-sign records. We further extracted immunocompromised status, defined as the presence of organ transplantation, long-term corticosteroid use, or active immunosuppressant therapy. Pre-ICU healthcare exposure was assessed using pre-ICU hospitalization status, transfer history, and pre-ICU hospitalization duration. To preserve data granularity, quantitative variables—including age, APACHE-II score, pre-ICU hospitalization duration, and the total pathogen detection count—were maintained as continuous measures in the regression models.

Prior anti-infective therapy before BALF sampling was also re-extracted and categorized by timing and drug class. Specifically, we recorded documented systemic antibacterial, anti-tuberculosis, antifungal, or antiviral therapy within 48 h, 7 days, and 14 days before BALF sampling. In the primary multivariable models, prior anti-infective therapy was analyzed as a binary variable indicating any documented systemic anti-infective therapy within 7 days before BALF sampling, whereas detailed drug-class information across all exposure windows was summarized descriptively in the supplementary materials. In this expanded baseline categorization, broad-spectrum antibacterial exposure was explicitly defined as the receipt of carbapenems, piperacillin-tazobactam, cefoperazone-sulbactam, vancomycin, linezolid, polymyxins, tigecycline, or other anti-pseudomonal/anti-MRSA regimens prior to sample collection.

### Statistical analysis

All statistical analyses were performed using R software version 4.5.2. We summarized continuous variables using means and standard deviations or medians and interquartile ranges, depending on data distribution, and compared them using the Student’s *t*-test or Mann-Whitney *U*-test. Categorical variables were expressed as frequencies and percentages and compared using the *χ*^2^ test or Fisher’s exact test. For pathogen targets with fewer than four positive Ct readouts, raw Ct values and internal-control-adjusted relative signals were summarized descriptively using ranges.

Pairwise correlations between binary pathogen targets were evaluated to characterize pathogen co-occurrence patterns. ROC curve analysis was performed to evaluate the discrimination of total pathogen detection count for mortality, and the exploratory cutoff was determined using the maximum Youden index.

To evaluate factors associated with mortality, we constructed multivariable logistic regression models for in-hospital mortality and Cox proportional hazards regression models for 60 day overall survival. Given the limited number of mortality events, the primary multivariable models used a clinically prioritized adjustment strategy and included age, APACHE-II score, prior anti-infective therapy within 7 days before BALF sampling, pre-ICU hospitalization days, and total pathogen detection count. ICU length of stay was not included in the Cox models to avoid time-dependent bias.

Sensitivity analyses were performed using alternative pathogen detection burden definitions and covariate structures, including recalculating detection counts after excluding *Candida albicans*, additionally adjusting for immunocompromised status, restricting the cohort to non-immunocompromised individuals, and excluding detections with raw Ct values >35 from the detection count. Multivariable models were fitted using complete cases for the variables included in each model. A two-sided *P* < 0.05 was considered statistically significant.

### Conclusion

In this single-center, TB-enriched ICU cohort, a higher cumulative respiratory pathogen detection burden by BALF mPCR was associated with increased short-term mortality after adjustment for clinical severity, prior anti-infective therapy, and pre-ICU hospitalization exposure. Because molecular detection does not necessarily indicate active infection, especially for colonization-prone organisms, these findings should be interpreted as evidence that pathogen detection complexity may serve as an early risk-stratification signal rather than as a direct therapeutic target. Prospective multicenter studies incorporating adjudicated infection status, antimicrobial exposure, host immune status, and semi-quantitative or quantitative pathogen-load metrics are needed to validate whether this metric can be integrated into ICU prognostic models.

## Supplementary Material

Reviewer comments

## Data Availability

The data sets analyzed in this study are not publicly available because they contain de-identified retrospective clinical laboratory records that are subject to institutional data governance and privacy restrictions. Requests to access these data sets should be directed to Yi Ren, yiren_whph@163.com. All data were anonymously collected and stored on a secured database. The data sets used during the current study are available from the corresponding author on reasonable request.

## SUPPLEMENTAL MATERIAL

Figure S1 spectrum.00865-26-s0001.tif Sensitivity and exploratory survival analyses stratified by alternative pathogen detection thresholds, both including and excluding *Candida albicans*.

Supplemental Material spectrum.00865-26-s0002.docx Tables S1 to S10; legend for Fig. S1.

OPEN PEER REVIEW reviewer-comments.pdf An accounting of the reviewer comments and feedback.

ASM does not own the copyrights to Supplemental Material that may be linked to, or accessed through, an article. The authors have granted ASM a non-exclusive, world-wide license to publish the Supplemental Material files. Please contact the corresponding author directly for reuse.
