# Supplementary material for: Cumulative respiratory pathogen detection burden by multiplex PCR is associated with mortality in a TB-enriched ICU cohort
Source: Microbiol Spectr. 2026 Jul 22;14(9):e00865-26. doi: 10.1128/spectrum.00865-26 (PMC13532216; doi:10.1128/spectrum.00865-26)
Supplement: Supplementary file 1 [file reviewer-comments.pdf]

AMERICAN SOCIETY FOR MICROBIOLOGY | Microbiology Spectrum

Cumulative Respiratory Pathogen Detection Burden by Multiplex PCR Is Associated With Mortality in a TB-Enriched ICU Cohort

Jurong Yan, Qian He, Lihui Zhao, Xunfei Yi, Aojia Wei, Ning Meng, and Yi Ren

Corresponding Author(s): Yi Ren, Wuhan Pulmonary Hospital

Review Timeline:

Submission Date:	March 10, 2026
Editorial Decision:	May 4, 2026
Revision Received:	May 28, 2026
Accepted:	July 1, 2026

Editor: Fei Chen

Reviewer(s): Disclosure of reviewer identity is with reference to reviewer comments included in decision letter(s). The following individuals involved in review of your submission have agreed to reveal their identity: Liya Yue (Reviewer #1); Xia Yu (Reviewer #2)

Transaction Report:

(Note: With the exception of the correction of typographical or spelling errors that could be a source of ambiguity, letters and reports are not edited. The original formatting of letters and referee reports may not be reflected in this compilation.)

DOI: https://doi.org/10.1128/spectrum.00865-26

Re: Spectrum00865-26 (**Cumulative Respiratory Pathogen Burden Detected by Multiplex PCR Predicts Mortality in a TB-Enriched ICU Cohort**)

Dear Dr. Yi Ren:

Thank you for the privilege of reviewing your work. Below you will find my comments, instructions from the Spectrum editorial office, and the reviewer comments.

Please return the manuscript within 60 days; if you cannot complete the modification within this time period, please contact me. If you do not wish to modify the manuscript and prefer to submit it to another journal, notify me immediately so that the manuscript may be formally withdrawn from consideration by Spectrum.

Revision Guidelines
To submit your modified manuscript, log into the submission site at https://spectrum.msubmit.net/cgi-bin/main.plex. Go to Author Tasks and click the appropriate manuscript title to begin. The information you entered when you first submitted the paper will be displayed; update this as necessary. Note the following requirements:

• Upload point-by-point responses to the issues raised by the reviewers in a file named "Response to Reviewers," NOT in your cover letter.
• Upload a compare copy of the manuscript (without figures) as a "Marked-Up Manuscript" file.
• Upload a clean .DOC/.DOCX version of the revised manuscript and remove the previous version.
• Each figure must be uploaded as a separate, editable, high-resolution file (TIFF or EPS preferred), and any multipanel figures must be assembled into one file.
• Any supplemental material intended for posting by ASM should be uploaded with their legends separate from the main manuscript. You can combine all supplemental material into one file (preferred) or split it into a maximum of 10 files with all associated legends included.

For complete guidelines on revision requirements, see our Submission and Review Process webpage. Submission of a paper that does not conform to guidelines may delay acceptance of your manuscript.

Data availability: ASM policy requires that data be available to the public upon online posting of the article, so please verify all links to sequence records, if present, and make sure that each number retrieves the full record of the data. If a new accession number is not linked or a link is broken, provide Spectrum production staff with the correct URL for the record. If the accession numbers for new data are not publicly accessible before the expected online posting of the article, publication may be delayed; please contact production staff (Spectrum@asmusa.org) immediately with the expected release date.

Publication Fees: For information on publication fees and which article types are subject to charges, visit our website. If your manuscript is accepted for publication and any fees apply, you will be contacted separately about payment during the production process; please follow the instructions in that e-mail. Arrangements for payment must be made before your article is published.

ASM Membership: Corresponding authors may join or renew ASM membership to obtain discounts on publication fees. Need to upgrade your membership level? Please contact Customer Service at Service@asmusa.org.

The ASM Journals program strives for constant improvement in our submission and publication process. Please tell us how we can improve your experience by taking this quick Author Survey.

Thank you for submitting your paper to Spectrum.

Sincerely,
Fei Chen
Editor
Microbiology Spectrum

Reviewer #1 (Comments for the Author):

This study examines the prognostic value of cumulative respiratory pathogen burden in a TB-enriched ICU cohort using multiplex PCR (mPCR). The focus on microbial complexity rather than single pathogens is clinically meaningful. However, several methodological and statistical issues, particularly related to confounding and model stability, need to be addressed before the conclusions can be supported.

1.Figure 5 legend: The authors state that "all models exclude the APACHE-II score to maintain a larger analytical sample size"

(Lines 731-732). Given that APACHE-II is a core predictor of ICU mortality, excluding it undermines the claim of independent association. The models should include APACHE-II, even at the cost of sample size, to show that pathogen burden is not simply reflecting underlying disease severity.

2.The manuscript does not account for prior or ongoing antimicrobial therapy. Such treatments can substantially affect both mPCR results and patient outcomes. This should be adjusted for in the analysis, or at least clearly acknowledged as a major limitation when interpreting pathogen burden.

3.Results, Lines 183-184: The high detection rate (38.5%) likely reflects colonization rather than true infection. Treating all detected organisms equally in the total count oversimplifies their clinical relevance. A sensitivity analysis excluding common colonizers would help determine whether the observed association with mortality is driven by true co-infection or by overall microbial diversity.

4.Figure 4: The survival curves suggest a threshold effect rather than a linear relationship. The authors should consider ROC analysis to identify an optimal cut-off value for pathogen count.

5.Table 5: The current correlation coefficients are difficult to interpret in isolation. A co-occurrence heat map would improve visualization of pathogen clustering patterns and make the results more intuitive.

6.Scientific names of microorganisms should be italicized throughout, and punctuation should be used consistently according to journal style (Line 438). Please review the entire manuscript and correct these issues accordingly.

Reviewer #2 (Comments for the Author):

The manuscript investigates the association between cumulative pathogen detection using multiplex PCR (mPCR) and clinical outcomes in critically ill patients with suspected tuberculosis or complex respiratory infections, and reports several observations regarding pathogen co-detection patterns. The study is well structured and addresses a clinically relevant topic; however, there are important methodological and interpretational limitations that need to be addressed before the conclusions can be fully supported. Below, I provide specific comments organised by the main issues of the manuscript.
1. In the Methods section, the authors appropriately acknowledge that "detection" does not equate to "infection." However, in the Results interpretation and prognostic analyses, the high detection rates of Candida albicans (38.5%) and various viruses are directly associated with mortality. This represents a major methodological challenge of the study. The authors are encouraged to stratify detection results in conjunction with clinical signs of infection, or to perform sensitivity analyses (e.g., excluding cases in which only microorganisms with low pathogenic potential are detected), in order to assess whether the core conclusions remain robust.
2. The central finding of this study is that "cumulative pathogen detection burden" is an independent prognostic factor for in-hospital mortality. However, the Discussion also acknowledges that a high burden may reflect underlying conditions such as host immune exhaustion or microbiome dysbiosis. The current analyses do not sufficiently distinguish whether polymicrobial infection per se directly increases the risk of death, or whether a high detection burden merely serves as a marker of underlying disease severity (e.g., more profound immunosuppression or a longer prior hospitalization history).
The authors are encouraged to strengthen causal inference by considering additional approaches, such as instrumental variable analysis, sensitivity analyses (e.g., excluding patients with early mortality), or more comprehensive adjustment for confounders, including pre-ICU antibiotic exposure and baseline immune status. Otherwise, the current conclusions may be prone to overinterpretation as implying that reducing pathogen burden alone would improve outcomes, which warrants more cautious framing.
3. In the logistic and Cox regression models, the authors adjusted for age and APACHE II score, but several key potential confounders were not considered: (a) immunosuppression status (e.g., solid organ transplantation, long-term corticosteroid or immunosuppressant use, HIV infection, and neutropenia), which is both a major risk factor for polymicrobial infection and a strong predictor of mortality, and is likely strongly correlated with total pathogen count; (b) prior antibiotic exposure and pre-admission length of hospitalization, as prolonged prior hospitalization and broader antibiotic use can directly shape the BALF microbiological profile (e.g., selection of resistant bacteria and fungi) and are also associated with worse outcomes.
Without adjustment for these factors, the independent prognostic effect of total pathogen burden may be substantially overestimated. The authors are therefore encouraged to include these covariates where feasible, or to perform propensity score matching or weighting analyses.
4. The study population is enriched for patients with suspected tuberculosis or complex infections, which may limit the applicability of the findings to general ICU populations. This should be more explicitly discussed in terms of external validity.
5. In Conclusions, the authors propose integrating cumulative pathogen burden into early risk stratification models to identify ultra-high-risk patients who may require more aggressive interventions. However, given the single-center retrospective design, the unresolved distinction between detection and infection, and the lack of adjustment for several key confounders, such practice-oriented recommendations appear premature. The authors are advised to temper this statement, for example, by suggesting that future prospective studies are needed to validate whether pathogen burden can be incorporated into risk prediction models, rather than making direct clinical recommendations.
6. The current analysis primarily relies on qualitative detection (presence/absence) of pathogens, without incorporating available

semi-quantitative measures such as Ct values. Integrating pathogen load information, if available, could further enhance the clinical interpretability of the findings.

This study examines the prognostic value of cumulative respiratory pathogen burden in a TB-enriched ICU cohort using multiplex PCR (mPCR). The focus on microbial complexity rather than single pathogens is clinically meaningful. However, several methodological and statistical issues, particularly related to confounding and model stability, need to be addressed before the conclusions can be supported.

1. Figure 5 legend: The authors state that “all models exclude the APACHE-II score to maintain a larger analytical sample size” (Lines 731–732). Given that APACHE-II is a core predictor of ICU mortality, excluding it undermines the claim of independent association. The models should include APACHE-II, even at the cost of sample size, to show that pathogen burden is not simply reflecting underlying disease severity.

2. The manuscript does not account for prior or ongoing antimicrobial therapy. Such treatments can substantially affect both mPCR results and patient outcomes. This should be adjusted for in the analysis, or at least clearly acknowledged as a major limitation when interpreting pathogen burden.

3. Results, Lines 183–184: The high detection rate (38.5%) likely reflects colonization rather than true infection. Treating all detected organisms equally in the total count oversimplifies their clinical relevance. A sensitivity analysis excluding common colonizers would help determine whether the observed association with mortality is driven by true co-infection or by overall microbial diversity.

4. Figure 4: The survival curves suggest a threshold effect rather than a linear relationship. The authors should consider ROC analysis to identify an optimal cut-off value for pathogen count.

5. Table 5: The current correlation coefficients are difficult to interpret in isolation. A co-occurrence heat map would improve visualization of pathogen clustering patterns and make the results more intuitive.

6. Scientific names of microorganisms should be italicized throughout, and punctuation should be used consistently according to journal style (Line 438). Please review the entire manuscript and correct these issues accordingly.

Response to Reviewers

Dear Editor and Reviewers,

We sincerely thank the reviewers for their constructive and insightful comments. We have substantially revised the manuscript to address the concerns regarding confounding, model stability, interpretation of molecular detection versus active infection, colonization-prone organisms, semi-quantitative Ct information, and external validity. In particular, we re-extracted additional clinical variables from the electronic medical records, completed APACHE-II scoring for all 130 patients, incorporated prior anti-infective therapy and pre-ICU hospitalization exposure into the primary analyses, extracted baseline immunocompromised status for sensitivity analyses, and performed several sensitivity analyses using alternative definitions of pathogen detection burden. We also tempered the language throughout the manuscript to avoid implying a direct causal effect of pathogen detection burden on mortality.

Reviewer #1:

Comment 1

Figure 5 legend: The authors state that "all models exclude the APACHE-II score to maintain a larger analytical sample size" (Lines 731-732). Given that APACHE-II is a core predictor of ICU mortality, excluding it undermines the claim of independent association. The models should include APACHE-II, even at the cost of sample size, to show that pathogen burden is not simply reflecting underlying disease severity.

Response:

We agree with the reviewer that APACHE-II is a core severity indicator in ICU mortality prediction and should be included in the primary multivariable models. In the original submission, APACHE-II data were incomplete for 39 patients, and the model excluding APACHE-II was presented in the main figure to preserve sample size. We recognize that this approach weakened the claim of independence from disease severity.

To address this issue, we carefully re-reviewed the electronic medical records, nursing records, laboratory data, arterial blood gas results, and vital-sign records, and completed APACHE-II scoring for all 130 patients. We then rebuilt the primary logistic regression and Cox proportional hazards models with APACHE-II included as a mandatory covariate.

In the revised manuscript, Figure 5 has been replaced with APACHE-II-adjusted models. The updated logistic and Cox models included age, APACHE-II score, prior anti-infective therapy within 7 days before BALF sampling, pre-ICU hospitalization days, and total pathogen detection count. Total pathogen detection count remained associated with in-hospital mortality in the logistic model (adjusted OR = 1.55, 95% CI: 1.14–2.16, P = 0.007) and with 60-day mortality hazard in the Cox model (adjusted HR = 1.42, 95% CI: 1.11–1.82, P = 0.006). The previous Figure 5 legend statement that APACHE-II was excluded to preserve sample size has been removed.

Comment 2

The manuscript does not account for prior or ongoing antimicrobial therapy. Such treatments can substantially affect both mPCR results and patient outcomes. This should be adjusted for in the analysis, or at least clearly acknowledged as a major limitation when interpreting pathogen burden.

Response:

We agree. Prior anti-infective therapy is an important potential confounder because it may influence both respiratory pathogen detection and clinical outcomes. In the revised analysis, we systematically extracted anti-infective exposure before BALF sampling for all 130 patients, including exposure within 48 hours, 7 days, and 14 days before sampling. We further categorized anti-infective therapy by class, including antibacterial agents, anti-tuberculosis therapy, antifungal therapy, antiviral therapy, and broad-spectrum antibacterial exposure. Broad-spectrum exposure was defined as receipt of carbapenems, piperacillin–tazobactam, cefoperazone–sulbactam, vancomycin or linezolid, polymyxins, tigecycline, or other anti-pseudomonal/anti-MRSA regimens before BALF sampling.

We added prior anti-infective therapy to the baseline characteristics table and included prior anti-infective therapy within 7 days before BALF sampling in the revised multivariable logistic and Cox models. Detailed exposure windows and drug-class information are summarized in Supplementary Table 1. We also acknowledged in the Discussion that detailed outpatient anti-infective regimens were incompletely documented in some patients and that residual confounding remains possible.

Comment 3

Results, Lines 183-184: The high detection rate (38.5%) likely reflects colonization rather than true infection. Treating all detected organisms equally in the total count oversimplifies their clinical relevance. A sensitivity analysis excluding common colonizers would help determine whether the observed association with mortality is driven by true co-infection or by overall microbial diversity.

Response:

We appreciate this important point. The reviewer is referring to the high detection rate of Candida albicans, which was the predominant “other pathogen” target in the original manuscript, detected in 50 of 130 patients (38.5%). We agree that respiratory Candida detection frequently reflects airway colonization rather than invasive infection and that treating all detected targets equally may oversimplify clinical interpretation.

We therefore performed additional sensitivity analyses using alternative pathogen-burden definitions and covariate structures. Specifically, we evaluated the association between pathogen detection count and outcomes both with and without Candida albicans inclusion across multiple scenarios: (1) the main multivariable model adjusted for age, APACHE-II score, prior anti-infective therapy within 7 days before BALF sampling, and pre-ICU hospitalization days; (2) a parsimonious model adjusted for age and APACHE-II score; and (3) a model further adjusted for immunocompromised status. To further address potential confounding from baseline host immune status, we also repeated these analyses after excluding immunocompromised patients.

Across these sensitivity models, the association between pathogen detection burden and mortality remained directionally consistent. After excluding Candida albicans from the detection count in the main adjusted model, the detection count remained associated with in-hospital mortality (adjusted OR = 1.57, 95% CI: 1.12–2.24, P = 0.010) and with 60-day mortality hazard in the Cox model (adjusted HR = 1.48, 95% CI: 1.13–1.94, P = 0.005). In the non-immunocompromised sub-cohort, the pathogen detection counts excluding Candida albicans also remained associated with outcomes (adjusted OR = 1.54, 95% CI: 1.11–2.21, P = 0.013; adjusted HR = 1.53, 95% CI: 1.16–2.02, P = 0.002). These sensitivity analyses are now summarized in Supplementary Table 9.

We revised the Results and Discussion to emphasize that the metric should be interpreted as “pathogen detection burden” or a microbial complexity signal, rather than as a direct measure of active polymicrobial infection.

Comment 4

Figure 4: The survival curves suggest a threshold effect rather than a linear relationship. The authors should consider ROC analysis to identify an optimal cut-off value for pathogen count.

Response:

We thank the reviewer for this helpful suggestion. We agree that the original Kaplan–Meier curves suggested a threshold-like relationship and that presenting several empirical thresholds could be difficult to interpret. Following the reviewer’s recommendation, we performed ROC analysis to identify an exploratory threshold for total pathogen detection count in predicting in-hospital mortality.

The Youden-derived threshold was 3.5, corresponding to a clinically interpretable grouping of >3 versus ≤3 detected targets. The AUC was 0.715 (95% CI: 0.586 – 0.844), with a sensitivity of 60.0% and a specificity of 72.7%. We revised Figure 4 accordingly: the ROC curve is now shown in Fig. 4B, and the Kaplan–Meier curve using this ROC-derived threshold is shown in Fig. 4C. The previous exploratory Kaplan–Meier curves using other empirical thresholds were moved to Fig. S1. Because of the single-center retrospective design and limited number of deaths, this threshold is described as exploratory rather than as a clinical

decision cutoff.

Comment 5

Table 5: The current correlation coefficients are difficult to interpret in isolation. A co-occurrence heat map would improve visualization of pathogen clustering patterns and make the results more intuitive.

Response:

We thank the reviewer for this helpful suggestion. We agree that pairwise correlation coefficients are difficult to interpret when presented only as a numerical table. In the revised manuscript, we optimized Fig. 2B as a correlation-based co-occurrence heatmap based on binary pathogen detection profiles across all BALF samples. The revised heatmap displays only the triangular matrix to reduce redundancy, uses a red-blue color scale to indicate positive and negative correlations, and marks statistically significant associations with asterisks.

We also revised the Results text to describe the co-occurrence structure shown in Fig. 2B and its relationship to the detailed numerical results in Table 5. Table 5 has been retained to provide the exact correlation coefficients, co-detected sample counts, and P values, whereas the revised Fig. 2B provides a more intuitive overview of pathogen co-occurrence patterns.

Comment 6

Scientific names of microorganisms should be italicized throughout, and punctuation should be used consistently according to journal style (Line 438). Please review the entire manuscript and correct these issues accordingly.

Response:

We thank the reviewer for identifying this formatting issue. We reviewed the entire manuscript and italicized genus and species names throughout the text, tables, and figure legends where applicable. We also reviewed punctuation and formatting consistency according to journal style.

Reviewer #2 :

Comment 1

In the Methods section, the authors appropriately acknowledge that "detection" does not equate to "infection." However, in the Results interpretation and prognostic analyses, the high detection rates of Candida albicans (38.5%) and various viruses are directly associated with mortality. This represents a major methodological challenge of the study. The authors are encouraged to stratify detection results in conjunction with clinical signs of infection, or to perform sensitivity analyses (e.g., excluding cases in which only microorganisms with low pathogenic potential are detected), in order to assess whether the core conclusions remain robust.

Response:

We thank the reviewer for this important comment. We agree that molecular detection does not necessarily indicate active infection and that this distinction should be maintained consistently throughout the manuscript. Although the original Introduction and Methods acknowledged that a positive nucleic acid signal requires clinical contextualization, we agree that the original Results and Discussion did not sufficiently distinguish “molecular detection” from “active infection.”

To address this concern, we revised the terminology throughout the manuscript and consistently used “detection,” “co-detection,” and “detection burden” rather than “infection burden” unless clinically justified. We also performed additional sensitivity analyses using alternative pathogen-burden definitions. First, to evaluate the impact of potential airway colonizers, we recalculated the pathogen detection count after excluding Candida albicans, the predominant “other pathogen” target, and repeated the multivariable regression analyses in the overall cohort and in the non-immunocompromised sub-cohort. Second, to assess whether weaker amplification signals near the positivity cutoff influenced the findings, we performed an additional sensitivity analysis excluding detections with raw Ct values >35.

The association between pathogen detection burden and mortality remained directionally consistent across these sensitivity

analyses. After excluding Candida albicans from the total count in the main adjusted model, pathogen detection burden remained significantly associated with in-hospital mortality (adjusted OR = 1.57, 95% CI: 1.12–2.24, P = 0.010) and with 60-day mortality hazard in the Cox model (adjusted HR = 1.48, 95% CI: 1.13–1.94, P = 0.005). This association also remained significant after excluding detections with raw Ct values >35 (Model S8 in Table S9: adjusted OR = 1.48, 95% CI: 1.09–2.06, P = 0.015; adjusted HR = 1.41, 95% CI: 1.09–1.82, P = 0.009). These sensitivity analyses are now presented in Supplementary Table 9.

We also revised the Discussion to emphasize that detection of Candida albicans or respiratory viruses in BALF should be interpreted in the clinical context and should not be assumed to represent active invasive infection without supportive clinical evidence.

Comment 2

The central finding of this study is that "cumulative pathogen detection burden" is an independent prognostic factor for in-hospital mortality. However, the Discussion also acknowledges that a high burden may reflect underlying conditions such as host immune exhaustion or microbiome dysbiosis. The current analyses do not sufficiently distinguish whether polymicrobial infection per se directly increases the risk of death, or whether a high detection burden merely serves as a marker of underlying disease severity (e.g., more profound immunosuppression or a longer prior hospitalization history).

The authors are encouraged to strengthen causal inference by considering additional approaches, such as instrumental variable analysis, sensitivity analyses (e.g., excluding patients with early mortality), or more comprehensive adjustment for confounders, including pre-ICU antibiotic exposure and baseline immune status. Otherwise, the current conclusions may be prone to overinterpretation as implying that reducing pathogen burden alone would improve outcomes, which warrants more cautious framing.

Response:

We agree with the reviewer that our retrospective observational study cannot establish that a higher pathogen detection burden directly causes mortality. The original Discussion already proposed that a high mPCR target count may serve as a surrogate marker for host immune exhaustion, impaired airway clearance, or microbiome dysbiosis. However, we agree that this point should be strengthened and that causal language should be tempered.

We made several revisions to address this concern. First, we expanded clinical data extraction and confounder adjustment. APACHE-II scores were completed for all 130 patients, and the revised primary logistic and Cox models now adjust for age, APACHE-II score, prior anti-infective therapy within 7 days before BALF sampling, and pre-ICU hospitalization days. Baseline immunocompromised status was additionally extracted and incorporated into sensitivity analyses.

Second, to assess whether the association was mainly explained by baseline immunocompromised status, we performed targeted sensitivity analyses after excluding immunocompromised patients, defined as those with organ transplantation, long-term corticosteroid use, or active immunosuppressant therapy (Models S6 and S7 in Table S9). Within this non-immunocompromised sub-cohort, the association between pathogen detection count and mortality remained directionally consistent and statistically significant. Specifically, the adjusted OR for in-hospital mortality was 1.57 (95% CI: 1.12–2.24, P = 0.010) for the total count (Model S6), and 1.54 (95% CI: 1.11–2.21, P = 0.013) when Candida albicans was excluded (Model S7). The Cox analyses showed similar findings for 60-day mortality hazard (Model S6: HR = 1.47; Model S7: HR = 1.53; both P < 0.01).

Third, we reviewed the early mortality pattern in the cohort. No patient died within the first 48 hours after ICU admission, and the earliest death occurred on day 3 after ICU admission. Therefore, immediate early mortality was not present in the analytic cohort.

Finally, we considered instrumental-variable analysis, as suggested by the reviewer. However, we did not identify a clinically valid instrument that would plausibly influence pathogen detection burden without also directly affecting mortality or baseline illness severity. Therefore, we did not perform instrumental-variable analysis, as using an invalid instrument could produce misleading inference. Instead, we strengthened confounder adjustment and sensitivity analyses and explicitly tempered causal language throughout the Abstract, Discussion, and Conclusion. In the revised manuscript, pathogen detection burden is described

as an early risk-stratification signal rather than as evidence of a direct causal therapeutic target.

Comment 3

In the logistic and Cox regression models, the authors adjusted for age and APACHE II score, but several key potential confounders were not considered: (a) immunosuppression status (e.g., solid organ transplantation, long-term corticosteroid or immunosuppressant use, HIV infection, and neutropenia), which is both a major risk factor for polymicrobial infection and a strong predictor of mortality, and is likely strongly correlated with total pathogen count; (b) prior antibiotic exposure and pre-admission length of hospitalization, as prolonged prior hospitalization and broader antibiotic use can directly shape the BALF microbiological profile (e.g., selection of resistant bacteria and fungi) and are also associated with worse outcomes.

Without adjustment for these factors, the independent prognostic effect of total pathogen burden may be substantially overestimated. The authors are therefore encouraged to include these covariates where feasible, or to perform propensity score matching or weighting analyses.

Response:

We agree with the reviewer that immunocompromised status, prior anti-infective exposure, and pre-ICU hospitalization exposure are important potential confounders that may influence both BALF pathogen detection profiles and clinical outcomes. To address this concern, we re-extracted these variables from the electronic medical records for all 130 patients.

Immunocompromised status was defined as the presence of solid organ transplantation, long-term corticosteroid use, or active immunosuppressant therapy. No patient in our cohort had documented HIV infection or neutropenia. Prior anti-infective exposure was extracted for 48 hours, 7 days, and 14 days before BALF sampling and was categorized by drug class, including antibacterial, anti-tuberculosis, antifungal, and antiviral therapy. Broad-spectrum antibacterial exposure was also summarized when specific regimens were available. Pre-ICU healthcare exposure was assessed using pre-ICU hospitalization status, transfer history, and pre-ICU hospitalization duration.

These variables have been added to the revised baseline table and mortality-stratified clinical table (Table 1 and Table 12), and detailed exposure windows and drug-class information are provided in Supplementary Table 1.

Given the sample size of 130 patients and the limited number of mortality events, we did not include all newly extracted covariates simultaneously in a single multivariable model, because this could increase the risk of overfitting and unstable estimates. For the same reason, we did not perform propensity-score matching or weighting, which would have reduced the effective sample size and may have produced unstable weights in this small cohort. Instead, we used a clinically prioritized modeling strategy combined with targeted sensitivity analyses.

Specifically, the revised primary logistic and Cox models now adjust for age, APACHE-II score, prior anti-infective therapy within 7 days before BALF sampling, and pre-ICU hospitalization days, in addition to total pathogen detection count (Fig. 5). To further address the potential confounding effect of baseline host immune status, we performed additional sensitivity analyses that included immunocompromised status as a covariate and analyses that excluded immunocompromised patients entirely. These results are reported in Supplementary Table 9. Across these models, the association between pathogen detection burden and mortality remained directionally consistent.

Comment 4

The study population is enriched for patients with suspected tuberculosis or complex infections, which may limit the applicability of the findings to general ICU populations. This should be more explicitly discussed in terms of external validity.

Response:

We agree with the reviewer that the TB-enriched and clinically selected nature of our cohort limits the generalizability of the findings to broader ICU populations. The original manuscript stated that the microbial ecology of our cohort may differ from unselected ICU pneumonia populations because of targeted enrollment criteria and the high pre-test probability of tuberculosis or complex infection. We have expanded this section to make the external-validity limitation more explicit.

In the revised Discussion, we now state that the findings should not be generalized to all ICU pneumonia patients, lower-risk pneumonia cohorts, non-TB-enriched settings, or centers with substantially different antimicrobial ecology and testing practices. We also emphasize that this cohort represents a clinically selected, TB-enriched, complex respiratory infection population in which BALF mPCR was preferentially used for patients with suspected tuberculosis, suspected atypical pathogens, or poor response to empirical therapy. Accordingly, we tempered the wording in the Discussion and Conclusion to frame cumulative pathogen detection burden as a risk-stratification signal requiring prospective multicenter validation rather than as a broadly generalizable clinical decision tool.

Comment 5

In Conclusions, the authors propose integrating cumulative pathogen burden into early risk stratification models to identify ultra-high-risk patients who may require more aggressive interventions. However, given the single-center retrospective design, the unresolved distinction between detection and infection, and the lack of adjustment for several key confounders, such practice-oriented recommendations appear premature. The authors are advised to temper this statement, for example, by suggesting that future prospective studies are needed to validate whether pathogen burden can be incorporated into risk prediction models, rather than making direct clinical recommendations.

Response:

We agree with the reviewer. The original Conclusion stated that cumulative pathogen burden may help identify ultra-high-risk patients requiring more aggressive interventions. We agree that this wording was too practice-oriented given the single-center retrospective design, the distinction between molecular detection and active infection, and the potential for residual confounding. We have therefore revised the Abstract, Discussion, and Conclusion to avoid premature clinical recommendations and to frame pathogen detection burden as a risk-stratification signal requiring further validation.

The revised Conclusion now reads:

"In this single-center, TB-enriched ICU cohort, a higher cumulative respiratory pathogen detection burden by BALF mPCR was associated with increased short-term mortality after adjustment for clinical severity, prior anti-infective therapy, and pre-ICU hospitalization exposure. Because molecular detection does not necessarily indicate active infection, especially for colonization-prone organisms, these findings should be interpreted as evidence that pathogen detection complexity may serve as an early risk-stratification signal rather than as a direct therapeutic target. Prospective multicenter studies incorporating adjudicated infection status, antimicrobial exposure, host immune status, and semi-quantitative or quantitative pathogen-load metrics are needed to validate whether this metric can be integrated into ICU prognostic models."

Comment 6

The current analysis primarily relies on qualitative detection (presence/absence) of pathogens, without incorporating available semi-quantitative measures such as Ct values. Integrating pathogen load information, if available, could further enhance the clinical interpretability of the findings.

Response:

We thank the reviewer for this important suggestion. During revision, we retrieved the original PCR amplification data and confirmed that Ct values were available for a subset of target-assigned positive detections. We agree that Ct values can provide useful semi-quantitative context beyond qualitative presence/absence results. However, because this assay is a single-tube multiplex PCR assay coupled with melting-curve analysis, Ct values should not be interpreted as standardized target-specific quantitative pathogen-load measurements.

To address the reviewer's suggestion, we summarized all available target-assigned raw Ct values and internal-control-adjusted relative signals in Supplementary Table 10. Across 158 positive detections with available raw Ct values, the overall median raw Ct was 27.30 (IQR: 21.91 – 32.27). Among these detections, 101/158 (63.9%) had Ct values ≤30, 34/158 (21.5%) had Ct values between >30 and ≤35, and 23/158 (14.6%) had Ct values between >35 and ≤38. Where internal-control Ct values were

available, ΔCt was calculated as pathogen Ct minus internal-control Ct, and $2^{-\Delta Ct}$ was summarized as an exploratory internal-control-adjusted relative signal.

We also performed a Ct-based sensitivity analysis by excluding detections with raw Ct values >35 from the pathogen count. The association between pathogen detection burden and outcomes remained statistically significant after this filtering step (Model S8 in Table S9): the adjusted OR for in-hospital mortality was 1.48 (95% CI: 1.09–2.06, P = 0.015), and the adjusted HR for 60-day mortality hazard was 1.41 (95% CI: 1.09–1.82, P = 0.009).

Because Ct and $2^{-\Delta Ct}$ values in this assay were not designed or validated as standardized quantitative pathogen-load measurements, we did not replace the primary qualitative detection-count analysis. Instead, these Ct-based results are presented as exploratory semi-quantitative context and are interpreted cautiously.

Re: Spectrum00865-26R1 (**Cumulative Respiratory Pathogen Detection Burden by Multiplex PCR Is Associated With Mortality in a TB-Enriched ICU Cohort**)

Dear Dr. Yi Ren:

Your manuscript has been accepted, and I am forwarding it to the ASM production staff for publication. Your paper will first be checked to make sure all elements meet the technical requirements. ASM staff will contact you if anything needs to be revised before copyediting and production can begin. Otherwise, you will be notified when your proofs are ready to be viewed.

Data Availability: ASM policy requires that data be available to the public upon online posting of the article, so please verify all links to sequence records, if present, and make sure that each number retrieves the full record of the data. If a new accession number is not linked or a link is broken, provide production staff with the correct URL for the record. If the accession numbers for new data are not publicly accessible before the expected online posting of the article, publication may be delayed; please contact ASM production staff immediately with the expected release date.

Publication Fees: For information on publication fees and which article types have charges, please visit our website. We have partnered with Copyright Clearance Center (CCC) to collect author charges. If fees apply to your paper, you will receive a message from no-reply@copyright.com with further instructions. For questions related to paying charges through RightsLink, please contact CCC at ASM_Support@copyright.com or toll free at +1-877-622-5543. CCC makes every attempt to respond to all emails within 24 hours.

ASM Membership: Corresponding authors may join or renew ASM membership to obtain discounts on publication fees. Need to upgrade your membership level? Please contact Customer Service at Service@asmusa.org.

Embargo Policy: A press release may be issued as soon as the manuscript is posted on the Spectrum Latest Articles webpage. The corresponding author will receive an email with the subject line "ASM Journals Author Services Notification" when the article is available online.

The ASM Journals program strives for constant improvement in our submission and publication process. Please tell us how we can improve your experience by taking this quick Author Survey.

Thank you for submitting your paper to Spectrum.

Sincerely,
Fei Chen
Editor
Microbiology Spectrum

Reviewer #1 (Comments for the Author):

The authors have adequately addressed my previous concerns. The revised manuscript now includes APACHE-II in the main logistic and Cox models, which makes the conclusion more convincing. Prior anti-infective therapy was also added to the analysis, which improves the clinical interpretation. The sensitivity analyses excluding Candida albicans and high-Ct detections are helpful, because they reduce the concern that the result is mainly driven by colonization or low-level signals. The ROC analysis, revised survival curves, and co-occurrence heatmap also make the results clearer. Overall, the revision is satisfactory. I have no further major concerns.

Reviewer #2 (Comments for the Author):

I have no further comments or suggestions for the authors. The manuscript is technically sound and well presented.

The authors have adequately addressed my previous concerns. The revised manuscript now includes APACHE-II in the main logistic and Cox models, which makes the conclusion more convincing. Prior anti-infective therapy was also added to the analysis, which improves the clinical interpretation. The sensitivity analyses excluding *Candida albicans* and high-Ct detections are helpful, because they reduce the concern that the result is mainly driven by colonization or low-level signals. The ROC analysis, revised survival curves, and co-occurrence heatmap also make the results clearer. Overall, the revision is satisfactory. I have no further major concerns.
