# Supplementary material for: Cumulative respiratory pathogen detection burden by multiplex PCR is associated with mortality in a TB-enriched ICU cohort
Source: Microbiol Spectr. 2026 Jul 22;14(9):e00865-26. doi: 10.1128/spectrum.00865-26 (PMC13532216; doi:10.1128/spectrum.00865-26)
Supplement: Supplementary file 2 [file spectrum.00865-26-s0002.docx]

**Supplementary Table 1** Detailed clinical characteristics, prior anti-infective exposure, and hospitalization history of study patients

| Characteristics | All Patients (n = 130) |
| --- | --- |
| Prior broad-spectrum antibiotic use, n (%) | 57 (43.8) |
| Prior anti-infective therapy within 48 hours before BALF sampling, n (%) |  |
| Antibacterial | 34 (26.2) |
| Anti-TB | 4 (3.1) |
| Antifungal | 5 (3.8) |
| Antiviral | 1 (0.8) |
| Prior anti-infective therapy within 7 days before BALF sampling, n (%) |  |
| Antibacterial | 63 (48.5) |
| Anti-TB | 7 (5.4) |
| Antifungal | 7 (5.4) |
| Antiviral | 3 (2.3) |
| Prior anti-infective therapy within 14 days before BALF sampling, n (%) |  |
| Antibacterial | 92 (70.8) |
| Anti-TB | 8 (6.2) |
| Antifungal | 16 (12.3) |
| Antiviral | 3 (2.3) |
| Hospitalizations within 90 days before BALF sampling, n (%) |  |
| 0 | 28 (21.5) |
| 1 | 99 (76.2) |
| ≥2 | 3 (2.3) |
| Rehospitalizations within 90 days after BALF sampling, n (%) |  |
| 0 | 89 (68.5) |
| 1 | 34 (26.2) |
| ≥2 | 7 (5.4) |

**Supplementary Table 2** Most Frequent Triple-Target Co-detection Combinations Identified in BALF Samples

| Triple-target combination | Count, n |
| --- | --- |
| Kpn + MRS-mecA + MTB | 15 |
| Aba + MTB + Cal | 14 |
| Aba + Kpn + MTB | 12 |
| Kpn + MTB + Cal | 11 |
| MRS-mecA + MTB + Cal | 11 |
| Aba + MRS-mecA + MTB | 10 |
| MTB + Pae + Cal | 10 |
| Aba + Kpn + MRS-mecA | 8 |
| Aba + Kpn + Cal | 7 |
| Kpn + MTB + Pae | 7 |
| Aba + MRS-mecA + Cal | 6 |
| Aba + MTB + Pae | 6 |
| Aba + Pae + Cal | 6 |
| MRS-mecA + MTB + Pae | 6 |
| Aba + Hin + MTB | 5 |
| Aba + MRS-mecA + Pae | 5 |
| Hin + MRS-mecA + MTB | 5 |
| Hin + MTB + Pae | 5 |
| Hin + MTB + Cal | 5 |
| Hin + Pae + Cal | 5 |
| Kpn + MRS-mecA + Pae | 5 |
| Kpn + MRS-mecA + Cal | 5 |
| MRS-mecA + MTB + Spn | 5 |
| Hin + MTB + Spn | 4 |
| Kpn + Pae + Cal | 4 |
| MRS-mecA + MTB + Pji | 4 |
| MRS-mecA + Pae + Cal | 4 |
| MRS-mecA + Sau + Cal | 4 |
| MTB + Sau + Cal | 4 |
| Aba + Kpn + Pae | 3 |
| Hin + Kpn + MTB | 3 |
| Hin + MRS-mecA + Spn | 3 |
| Kpn + MTB + Spn | 3 |
| MRS-mecA + MTB + Sau | 3 |
| MRS-mecA + MTB + HRV | 3 |
| MTB + Pae + IFA | 3 |
| MTB + IFA + Cal | 3 |
| Aba + Hin + Kpn | 2 |
| Aba + Hin + MRS-mecA | 2 |
| Aba + Hin + Pae | 2 |
| Aba + Hin + Cal | 2 |
| Aba + Kpn + Mca | 2 |
| Aba + Mca + MTB | 2 |
| Aba + Mca + Pae | 2 |
| Aba + MRS-mecA + Spn | 2 |
| Aba + MTB + Spn | 2 |
| Aba + MTB + IFA | 2 |
| Aba + MTB + PIV | 2 |
| Aba + Pae + IFA | 2 |
| Aba + Pae + Lpn | 2 |
| Aba + PIV + Cal | 2 |
| Aba + RSV + Cal | 2 |
| Aba + SARS-CoV-2 + Cal | 2 |
| Hin + Kpn + MRS-mecA | 2 |
| Hin + MRS-mecA + Cal | 2 |
| Hin + Pae + Spn | 2 |
| Hin + Spn + Cal | 2 |
| Kpn + Mca + MTB | 2 |
| Kpn + MRS-mecA + Spn | 2 |
| Kpn + MRS-mecA + HRV | 2 |
| Kpn + MRS-mecA + PIV | 2 |
| Kpn + MTB + HRV | 2 |
| Kpn + MTB + PIV | 2 |
| Kpn + Pae + Spn | 2 |
| Mca + MRS-mecA + MTB | 2 |
| MRS-mecA + MTB + IFA | 2 |
| MRS-mecA + MTB + PIV | 2 |
| MRS-mecA + Pae + Sau | 2 |
| MRS-mecA + Sau + IFA | 2 |
| MRS-mecA + Spn + Cal | 2 |
| MRS-mecA + IFA + Cal | 2 |
| MRS-mecA + Cal + Pji | 2 |
| MTB + Pae + Spn | 2 |
| MTB + Pae + HRV | 2 |
| MTB + Sau + IFA | 2 |
| MTB + Spn + Cal | 2 |
| MTB + HRV + IFA | 2 |
| MTB + HRV + Cal | 2 |
| MTB + PIV + Cal | 2 |
| MTB + Cal + Pji | 2 |
| Pae + Sau + Cal | 2 |
| Sau + IFA + Cal | 2 |
| Aba + Hin + Spn | 1 |
| Aba + Hin + IFA | 1 |
| Aba + Kpn + Spn | 1 |
| Aba + Kpn + PIV | 1 |
| Aba + Mca + MRS-mecA | 1 |
| Aba + Mca + Spn | 1 |
| Aba + Mca + Cal | 1 |
| Aba + Mca + Lpn | 1 |
| Aba + Mca + Pji | 1 |
| Aba + MRS-mecA + Sau | 1 |
| Aba + MRS-mecA + HCoV | 1 |
| Aba + MRS-mecA + PIV | 1 |
| Aba + MRS-mecA + RSV | 1 |
| Aba + MRS-mecA + SARS-CoV-2 | 1 |
| Aba + MRS-mecA + Pji | 1 |
| Aba + MTB + RSV | 1 |
| Aba + MTB + SARS-CoV-2 | 1 |
| Aba + MTB + Lpn | 1 |
| Aba + MTB + Pji | 1 |
| Aba + Pae + Sau | 1 |
| Aba + Pae + Spn | 1 |
| Aba + Pae + HCoV | 1 |
| Aba + Pae + RSV | 1 |
| Aba + Pae + SARS-CoV-2 | 1 |
| Aba + Pae + Pji | 1 |
| Aba + Sau + RSV | 1 |
| Aba + Sau + Cal | 1 |
| Aba + IFA + Cal | 1 |
| Aba + SARS-CoV-2 + Pji | 1 |
| Aba + Cal + Lpn | 1 |
| Aba + Cal + Pji | 1 |
| Aba + Lpn + Pji | 1 |
| Hin + Kpn + Pae | 1 |
| Hin + Kpn + Spn | 1 |
| Hin + Kpn + Cal | 1 |
| Hin + MRS-mecA + Pae | 1 |
| Hin + MRS-mecA + Sau | 1 |
| Hin + MTB + Sau | 1 |
| Hin + MTB + IFA | 1 |
| Hin + Pae + Sau | 1 |
| Hin + Pae + IFA | 1 |
| Hin + Sau + Cal | 1 |
| Hin + IFA + Cal | 1 |
| Kpn + Mca + MRS-mecA | 1 |
| Kpn + Mca + Pae | 1 |
| Kpn + Mca + Spn | 1 |
| Kpn + Mca + Cal | 1 |
| Kpn + MRS-mecA + Pji | 1 |
| Kpn + MTB + Sau | 1 |
| Kpn + MTB + Pji | 1 |
| Kpn + Pae + HRV | 1 |
| Kpn + Pae + Pji | 1 |
| Kpn + Sau + Cal | 1 |
| Kpn + HRV + PIV | 1 |
| Kpn + HRV + Pji | 1 |
| Kpn + PIV + Cal | 1 |
| Mca + MRS-mecA + Pae | 1 |
| Mca + MRS-mecA + Spn | 1 |
| Mca + MRS-mecA + RSV | 1 |
| Mca + MRS-mecA + Pji | 1 |
| Mca + MTB + Pae | 1 |
| Mca + MTB + Spn | 1 |
| Mca + MTB + RSV | 1 |
| Mca + MTB + Cal | 1 |
| Mca + MTB + Pji | 1 |
| Mca + Pae + Spn | 1 |
| Mca + Pae + Lpn | 1 |
| Mca + Pae + Pji | 1 |
| Mca + RSV + Pji | 1 |
| Mca + Lpn + Pji | 1 |
| MRS-mecA + MTB + HCoV | 1 |
| MRS-mecA + MTB + RSV | 1 |
| MRS-mecA + MTB + SARS-CoV-2 | 1 |
| MRS-mecA + Pae + Spn | 1 |
| MRS-mecA + Pae + HCoV | 1 |
| MRS-mecA + Pae + HRV | 1 |
| MRS-mecA + Pae + RSV | 1 |
| MRS-mecA + Pae + Pji | 1 |
| MRS-mecA + Sau + HRV | 1 |
| MRS-mecA + Sau + RSV | 1 |
| MRS-mecA + Sau + Pji | 1 |
| MRS-mecA + HRV + IFA | 1 |
| MRS-mecA + HRV + PIV | 1 |
| MRS-mecA + HRV + Cal | 1 |
| MRS-mecA + HRV + Pji | 1 |
| MRS-mecA + IFA + Pji | 1 |
| MRS-mecA + PIV + Cal | 1 |
| MRS-mecA + RSV + Cal | 1 |
| MRS-mecA + RSV + Pji | 1 |
| MRS-mecA + SARS-CoV-2 + Cal | 1 |
| MRS-mecA + SARS-CoV-2 + Pji | 1 |
| MTB + Pae + Sau | 1 |
| MTB + Pae + Lpn | 1 |
| MTB + Pae + Pji | 1 |
| MTB + Sau + HRV | 1 |
| MTB + Sau + Pji | 1 |
| MTB + HCoV + SARS-CoV-2 | 1 |
| MTB + HRV + PIV | 1 |
| MTB + HRV + Pji | 1 |
| MTB + IFA + Pji | 1 |
| MTB + RSV + Cal | 1 |
| MTB + RSV + Pji | 1 |
| MTB + SARS-CoV-2 + Cal | 1 |
| MTB + SARS-CoV-2 + Pji | 1 |
| MTB + Cal + Lpn | 1 |
| Pae + Sau + RSV | 1 |
| Pae + Spn + Cal | 1 |
| Pae + HRV + Cal | 1 |
| Pae + HRV + Pji | 1 |
| Pae + IFA + Cal | 1 |
| Pae + RSV + Cal | 1 |
| Pae + SARS-CoV-2 + Cal | 1 |
| Pae + Cal + Lpn | 1 |
| Pae + Lpn + Pji | 1 |
| Sau + HRV + IFA | 1 |
| Sau + HRV + Cal | 1 |
| Sau + IFA + Pji | 1 |
| Sau + RSV + Cal | 1 |
| Sau + Cal + Pji | 1 |
| HRV + IFA + Cal | 1 |
| HRV + SARS-CoV-2 + Cal | 1 |
| IFA + Cal + Pji | 1 |
| SARS-CoV-2 + Cal + Pji | 1 |

**Supplementary Table 3** Most Frequent Quadruple-Target Co-detection Combinations Identified in BALF Samples

| Quadruple-target combination | Count, n |
| --- | --- |
| Aba + Kpn + MRS-mecA + MTB | 8 |
| Aba + Kpn + MTB + Cal | 7 |
| Kpn + MRS-mecA + MTB + Pae | 5 |
| Kpn + MRS-mecA + MTB + Cal | 5 |
| Aba + MRS-mecA + MTB + Cal | 4 |
| Hin + MTB + Pae + Cal | 4 |
| Aba + Kpn + MRS-mecA + Cal | 3 |
| Aba + Kpn + MTB + Pae | 3 |
| Aba + MTB + Pae + Cal | 3 |
| Hin + MRS-mecA + MTB + Spn | 3 |
| Kpn + MTB + Pae + Cal | 3 |
| MRS-mecA + MTB + Sau + Cal | 3 |
| Aba + Hin + Kpn + MTB | 2 |
| Aba + Hin + MRS-mecA + MTB | 2 |
| Aba + Hin + MTB + Pae | 2 |
| Aba + Hin + MTB + Cal | 2 |
| Aba + Hin + Pae + Cal | 2 |
| Aba + Kpn + Mca + MTB | 2 |
| Aba + Kpn + MRS-mecA + Pae | 2 |
| Aba + MRS-mecA + MTB + Pae | 2 |
| Aba + MRS-mecA + MTB + Spn | 2 |
| Aba + MRS-mecA + Pae + Cal | 2 |
| Aba + MTB + Pae + IFA | 2 |
| Aba + MTB + PIV + Cal | 2 |
| Hin + Kpn + MRS-mecA + MTB | 2 |
| Hin + MRS-mecA + MTB + Cal | 2 |
| Kpn + MRS-mecA + MTB + Spn | 2 |
| Kpn + MRS-mecA + MTB + HRV | 2 |
| Kpn + MRS-mecA + MTB + PIV | 2 |
| MRS-mecA + MTB + Pae + Cal | 2 |
| MRS-mecA + MTB + Sau + IFA | 2 |
| MRS-mecA + MTB + Spn + Cal | 2 |
| MRS-mecA + MTB + IFA + Cal | 2 |
| MRS-mecA + MTB + Cal + Pji | 2 |
| MRS-mecA + Pae + Sau + Cal | 2 |
| MRS-mecA + Sau + IFA + Cal | 2 |
| MTB + Sau + IFA + Cal | 2 |
| Aba + Hin + Kpn + MRS-mecA | 1 |
| Aba + Hin + Kpn + Pae | 1 |
| Aba + Hin + Kpn + Cal | 1 |
| Aba + Hin + MRS-mecA + Spn | 1 |
| Aba + Hin + MTB + Spn | 1 |
| Aba + Hin + MTB + IFA | 1 |
| Aba + Hin + Pae + IFA | 1 |
| Aba + Hin + IFA + Cal | 1 |
| Aba + Kpn + Mca + MRS-mecA | 1 |
| Aba + Kpn + Mca + Pae | 1 |
| Aba + Kpn + Mca + Spn | 1 |
| Aba + Kpn + Mca + Cal | 1 |
| Aba + Kpn + MRS-mecA + Spn | 1 |
| Aba + Kpn + MRS-mecA + PIV | 1 |
| Aba + Kpn + MTB + Spn | 1 |
| Aba + Kpn + MTB + PIV | 1 |
| Aba + Kpn + Pae + Spn | 1 |
| Aba + Kpn + Pae + Cal | 1 |
| Aba + Kpn + PIV + Cal | 1 |
| Aba + Mca + MRS-mecA + MTB | 1 |
| Aba + Mca + MRS-mecA + Pae | 1 |
| Aba + Mca + MRS-mecA + Spn | 1 |
| Aba + Mca + MTB + Pae | 1 |
| Aba + Mca + MTB + Spn | 1 |
| Aba + Mca + MTB + Cal | 1 |
| Aba + Mca + Pae + Spn | 1 |
| Aba + Mca + Pae + Lpn | 1 |
| Aba + Mca + Pae + Pji | 1 |
| Aba + Mca + Lpn + Pji | 1 |
| Aba + MRS-mecA + MTB + PIV | 1 |
| Aba + MRS-mecA + MTB + SARS-CoV-2 | 1 |
| Aba + MRS-mecA + MTB + Pji | 1 |
| Aba + MRS-mecA + Pae + Sau | 1 |
| Aba + MRS-mecA + Pae + Spn | 1 |
| Aba + MRS-mecA + Pae + HCoV | 1 |
| Aba + MRS-mecA + Pae + RSV | 1 |
| Aba + MRS-mecA + Sau + RSV | 1 |
| Aba + MRS-mecA + Sau + Cal | 1 |
| Aba + MRS-mecA + PIV + Cal | 1 |
| Aba + MRS-mecA + RSV + Cal | 1 |
| Aba + MRS-mecA + SARS-CoV-2 + Cal | 1 |
| Aba + MRS-mecA + SARS-CoV-2 + Pji | 1 |
| Aba + MRS-mecA + Cal + Pji | 1 |
| Aba + MTB + Pae + Spn | 1 |
| Aba + MTB + Pae + Lpn | 1 |
| Aba + MTB + IFA + Cal | 1 |
| Aba + MTB + RSV + Cal | 1 |
| Aba + MTB + SARS-CoV-2 + Cal | 1 |
| Aba + MTB + SARS-CoV-2 + Pji | 1 |
| Aba + MTB + Cal + Lpn | 1 |
| Aba + MTB + Cal + Pji | 1 |
| Aba + Pae + Sau + RSV | 1 |
| Aba + Pae + Sau + Cal | 1 |
| Aba + Pae + IFA + Cal | 1 |
| Aba + Pae + RSV + Cal | 1 |
| Aba + Pae + SARS-CoV-2 + Cal | 1 |
| Aba + Pae + Cal + Lpn | 1 |
| Aba + Pae + Lpn + Pji | 1 |
| Aba + Sau + RSV + Cal | 1 |
| Aba + SARS-CoV-2 + Cal + Pji | 1 |
| Hin + Kpn + MRS-mecA + Spn | 1 |
| Hin + Kpn + MTB + Pae | 1 |
| Hin + Kpn + MTB + Spn | 1 |
| Hin + Kpn + MTB + Cal | 1 |
| Hin + Kpn + Pae + Cal | 1 |
| Hin + MRS-mecA + MTB + Pae | 1 |
| Hin + MRS-mecA + MTB + Sau | 1 |
| Hin + MRS-mecA + Pae + Sau | 1 |
| Hin + MRS-mecA + Pae + Cal | 1 |
| Hin + MRS-mecA + Sau + Cal | 1 |
| Hin + MRS-mecA + Spn + Cal | 1 |
| Hin + MTB + Pae + Sau | 1 |
| Hin + MTB + Pae + Spn | 1 |
| Hin + MTB + Pae + IFA | 1 |
| Hin + MTB + Sau + Cal | 1 |
| Hin + MTB + Spn + Cal | 1 |
| Hin + MTB + IFA + Cal | 1 |
| Hin + Pae + Sau + Cal | 1 |
| Hin + Pae + Spn + Cal | 1 |
| Hin + Pae + IFA + Cal | 1 |
| Kpn + Mca + MRS-mecA + MTB | 1 |
| Kpn + Mca + MRS-mecA + Pae | 1 |
| Kpn + Mca + MRS-mecA + Spn | 1 |
| Kpn + Mca + MTB + Pae | 1 |
| Kpn + Mca + MTB + Spn | 1 |
| Kpn + Mca + MTB + Cal | 1 |
| Kpn + Mca + Pae + Spn | 1 |
| Kpn + MRS-mecA + MTB + Pji | 1 |
| Kpn + MRS-mecA + Pae + Spn | 1 |
| Kpn + MRS-mecA + Pae + HRV | 1 |
| Kpn + MRS-mecA + Pae + Cal | 1 |
| Kpn + MRS-mecA + Pae + Pji | 1 |
| Kpn + MRS-mecA + HRV + PIV | 1 |
| Kpn + MRS-mecA + HRV + Pji | 1 |
| Kpn + MRS-mecA + PIV + Cal | 1 |
| Kpn + MTB + Pae + Spn | 1 |
| Kpn + MTB + Pae + HRV | 1 |
| Kpn + MTB + Pae + Pji | 1 |
| Kpn + MTB + Sau + Cal | 1 |
| Kpn + MTB + HRV + PIV | 1 |
| Kpn + MTB + HRV + Pji | 1 |
| Kpn + MTB + PIV + Cal | 1 |
| Kpn + Pae + HRV + Pji | 1 |
| Mca + MRS-mecA + MTB + Pae | 1 |
| Mca + MRS-mecA + MTB + Spn | 1 |
| Mca + MRS-mecA + MTB + RSV | 1 |
| Mca + MRS-mecA + MTB + Pji | 1 |
| Mca + MRS-mecA + Pae + Spn | 1 |
| Mca + MRS-mecA + RSV + Pji | 1 |
| Mca + MTB + Pae + Spn | 1 |
| Mca + MTB + RSV + Pji | 1 |
| Mca + Pae + Lpn + Pji | 1 |
| MRS-mecA + MTB + Pae + Sau | 1 |
| MRS-mecA + MTB + Pae + Spn | 1 |
| MRS-mecA + MTB + Pae + HRV | 1 |
| MRS-mecA + MTB + Pae + Pji | 1 |
| MRS-mecA + MTB + Sau + HRV | 1 |
| MRS-mecA + MTB + Sau + Pji | 1 |
| MRS-mecA + MTB + HRV + IFA | 1 |
| MRS-mecA + MTB + HRV + PIV | 1 |
| MRS-mecA + MTB + HRV + Cal | 1 |
| MRS-mecA + MTB + HRV + Pji | 1 |
| MRS-mecA + MTB + IFA + Pji | 1 |
| MRS-mecA + MTB + PIV + Cal | 1 |
| MRS-mecA + MTB + RSV + Pji | 1 |
| MRS-mecA + MTB + SARS-CoV-2 + Cal | 1 |
| MRS-mecA + MTB + SARS-CoV-2 + Pji | 1 |
| MRS-mecA + Pae + Sau + RSV | 1 |
| MRS-mecA + Pae + HRV + Pji | 1 |
| MRS-mecA + Pae + RSV + Cal | 1 |
| MRS-mecA + Sau + HRV + IFA | 1 |
| MRS-mecA + Sau + HRV + Cal | 1 |
| MRS-mecA + Sau + IFA + Pji | 1 |
| MRS-mecA + Sau + RSV + Cal | 1 |
| MRS-mecA + Sau + Cal + Pji | 1 |
| MRS-mecA + HRV + IFA + Cal | 1 |
| MRS-mecA + IFA + Cal + Pji | 1 |
| MRS-mecA + SARS-CoV-2 + Cal + Pji | 1 |
| MTB + Pae + Sau + Cal | 1 |
| MTB + Pae + HRV + Cal | 1 |
| MTB + Pae + HRV + Pji | 1 |
| MTB + Pae + IFA + Cal | 1 |
| MTB + Pae + Cal + Lpn | 1 |
| MTB + Sau + HRV + IFA | 1 |
| MTB + Sau + HRV + Cal | 1 |
| MTB + Sau + IFA + Pji | 1 |
| MTB + Sau + Cal + Pji | 1 |
| MTB + HRV + IFA + Cal | 1 |
| MTB + IFA + Cal + Pji | 1 |
| MTB + SARS-CoV-2 + Cal + Pji | 1 |
| Pae + Sau + RSV + Cal | 1 |
| Sau + HRV + IFA + Cal | 1 |
| Sau + IFA + Cal + Pji | 1 |

**Supplementary Table 4** Selected Age-Stratified Detection Rates for Representative Single Targets and Co-detection Combinations (Data Used for Figure 3A)

| Pathogen detection | Age < 60, n(%) | 60 ≤ Age < 75, n(%) | Age ≥ 75, n(%) | p-value | Statistical test |
| --- | --- | --- | --- | --- | --- |
| MTB | 21 (67.7) | 34 (60.7) | 30 (69.8) | 0.612 | Chi-square test |
| Pae | 10 (32.3) | 19 (33.9) | 10 (23.3) | 0.492 | Chi-square test |
| Kpn | 10 (32.3) | 18 (32.1) | 10 (23.3) | 0.574 | Chi-square test |
| HRV | 3 (9.7) | 3 (5.4) | 2 (4.7) | 0.726 | Fisher's exact test |
| IFA | 3 (9.7) | 2 (3.6) | 3 (7.0) | 0.484 | Fisher's exact test |
| SARS-CoV-2 | 1 (3.2) | 3 (5.4) | 1 (2.3) | 0.845 | Fisher's exact test |
| Cal | 13 (41.9) | 18 (32.1) | 19 (44.2) | 0.428 | Chi-square test |
| Pji | 2 (6.5) | 1 (1.8) | 3 (7.0) | 0.385 | Fisher's exact test |
| Lpn | 0 (0.0) | 2 (3.6) | 1 (2.3) | 0.788 | Fisher's exact test |
| Aba | 6 (19.4) | 19 (33.9) | 10 (23.3) | 0.273 | Chi-square test |
| Spn | 2 (6.5) | 6 (10.7) | 2 (4.7) | 0.589 | Fisher's exact test |
| Hin+Spn | 2 (6.5) | 3 (5.4) | 0 (0.0) | 0.242 | Fisher's exact test |
| Mca+Pji | 0 (0.0) | 1 (1.8) | 1 (2.3) | 1 | Fisher's exact test |
| Sau+IFA | 0 (0.0) | 1 (1.8) | 1 (2.3) | 1 | Fisher's exact test |
| Kpn+MRS-mecA | 5 (16.1) | 7 (12.5) | 4 (9.3) | 0.703 | Fisher's exact test |
| Aba+Cal | 4 (12.9) | 10 (17.9) | 6 (14.0) | 0.856 | Fisher's exact test |
| MRS-mecA+MTB | 9 (29.0) | 9 (16.1) | 9 (20.9) | 0.361 | Chi-square test |
| Kpn+MRS-mecA+MTB | 5 (16.1) | 6 (10.7) | 4 (9.3) | 0.689 | Fisher's exact test |
| Aba+MTB+Cal | 3 (9.7) | 7 (12.5) | 4 (9.3) | 0.939 | Fisher's exact test |
| Aba+Kpn+MTB | 3 (9.7) | 6 (10.7) | 3 (7.0) | 0.874 | Fisher's exact test |
| Aba+Kpn+MRS-mecA+MTB | 2 (6.5) | 3 (5.4) | 3 (7.0) | 1 | Fisher's exact test |
| Aba+Kpn+MTB+Cal | 2 (6.5) | 3 (5.4) | 2 (4.7) | 1 | Fisher's exact test |
| Kpn+MRS-mecA+MTB+Pae | 3 (9.7) | 2 (3.6) | 0 (0.0) | 0.118 | Fisher's exact test |

**Supplementary Table 5** Selected Sex-Stratified Detection Rates for Representative Single Targets and Co-detection Combinations (Data Used for Figure 3B)

| Pathogen detection | Male, n(%) | Female, n(%) | *p*-value | Statistical test |
| --- | --- | --- | --- | --- |
| MTB | 69 (69.0) | 16 (53.3) | 0.173 | Chi-square test |
| Pae | 29 (29.0) | 10 (33.3) | 0.82 | Chi-square test |
| Kpn | 34 (34.0) | 4 (13.3) | 0.038 | Fisher's exact test |
| HRV | 6 (6.0) | 2 (6.7) | 1 | Fisher's exact test |
| IFA | 4 (4.0) | 4 (13.3) | 0.082 | Fisher's exact test |
| SARS-CoV-2 | 5 (5.0) | 0 (0.0) | 0.589 | Fisher's exact test |
| Cal | 35 (35.0) | 15 (50.0) | 0.205 | Chi-square test |
| Pji | 4 (4.0) | 2 (6.7) | 0.621 | Fisher's exact test |
| Lpn | 0 (0.0) | 3 (10.0) | 0.011 | Fisher's exact test |
| Aba | 26 (26.0) | 9 (30.0) | 0.843 | Chi-square test |
| Spn | 9 (9.0) | 1 (3.3) | 0.452 | Fisher's exact test |
| Hin+Spn | 5 (5.0) | 0 (0.0) | 0.589 | Fisher's exact test |
| Mca+Pji | 1 (1.0) | 1 (3.3) | 0.41 | Fisher's exact test |
| Sau+IFA | 0 (0.0) | 2 (6.7) | 0.052 | Fisher's exact test |
| Kpn+MRS-mecA | 14 (14.0) | 2 (6.7) | 0.359 | Fisher's exact test |
| Aba+Cal | 13 (13.0) | 7 (23.3) | 0.277 | Chi-square test |
| MRS-mecA+MTB | 21 (21.0) | 6 (20.0) | 1 | Chi-square test |
| Kpn+MRS-mecA+MTB | 13 (13.0) | 2 (6.7) | 0.518 | Fisher's exact test |
| Aba+MTB+Cal | 9 (9.0) | 5 (16.7) | 0.394 | Chi-square test |
| Aba+Kpn+MTB | 9 (9.0) | 3 (10.0) | 1 | Fisher's exact test |
| Aba+Kpn+MRS-mecA+MTB | 6 (6.0) | 2 (6.7) | 1 | Fisher's exact test |
| Aba+Kpn+MTB+Cal | 5 (5.0) | 2 (6.7) | 0.661 | Fisher's exact test |
| Kpn+MRS-mecA+MTB+Pae | 5 (5.0) | 0 (0.0) | 0.589 | Fisher's exact test |

**Supplementary Table 6** Selected APACHE-II–Stratified Detection Rates for Representative Single Targets and Co-detection Combinations (Data Used for Figure 3D)

| Pathogen detection | <20, n(%) | ≥20, n(%) | *p*-value | Statistical test |
| --- | --- | --- | --- | --- |
| MTB | 34 (57.6) | 51 (71.8) | 0.131 | Chi-square test |
| Pae | 17 (28.8) | 22 (31.0) | 0.939 | Chi-square test |
| Kpn | 16 (27.1) | 22 (31.0) | 0.773 | Chi-square test |
| HRV | 1 (1.7) | 7 (9.9) | 0.071 | Fisher's exact test |
| IFA | 2 (3.4) | 6 (8.5) | 0.291 | Fisher's exact test |
| SARS-CoV-2 | 4 (6.8) | 1 (1.4) | 0.176 | Fisher's exact test |
| Cal | 22 (37.3) | 28 (39.4) | 0.944 | Chi-square test |
| Pji | 1 (1.7) | 5 (7.0) | 0.22 | Fisher's exact test |
| Lpn | 1 (1.7) | 2 (2.8) | 1 | Fisher's exact test |
| Aba | 8 (13.6) | 27 (38.0) | 0.003 | Chi-square test |
| Spn | 8 (13.6) | 2 (2.8) | 0.042 | Fisher's exact test |
| Hin+Spn | 4 (6.8) | 1 (1.4) | 0.176 | Fisher's exact test |
| Mca+Pji | 0 (0.0) | 2 (2.8) | 0.5 | Fisher's exact test |
| Sau+IFA | 0 (0.0) | 2 (2.8) | 0.5 | Fisher's exact test |
| Kpn+MRS-mecA | 6 (10.2) | 10 (14.1) | 0.683 | Chi-square test |
| Aba+Cal | 4 (6.8) | 16 (22.5) | 0.015 | Fisher's exact test |
| MRS-mecA+MTB | 10 (16.9) | 17 (23.9) | 0.446 | Chi-square test |
| Kpn+MRS-mecA+MTB | 6 (10.2) | 9 (12.7) | 0.865 | Chi-square test |
| Aba+MTB+Cal | 2 (3.4) | 12 (16.9) | 0.02 | Fisher's exact test |
| Aba+Kpn+MTB | 3 (5.1) | 9 (12.7) | 0.223 | Fisher's exact test |
| Aba+Kpn+MRS-mecA+MTB | 2 (3.4) | 6 (8.5) | 0.291 | Fisher's exact test |
| Aba+Kpn+MTB+Cal | 1 (1.7) | 6 (8.5) | 0.126 | Fisher's exact test |
| Kpn+MRS-mecA+MTB+Pae | 3 (5.1) | 2 (2.8) | 0.658 | Fisher's exact test |

**Supplementary Table 7** Selected Comorbidity-Stratified Detection Rates for Representative Single Targets and Co-detection Combinations (Data Used for Figure 3C)

| Pathogen detection | Non-comorbidity, n(%) | Comorbidity, n(%) | *p*-value | Statistical test |
| --- | --- | --- | --- | --- |
| MTB | 45 (75.0) | 38 (55.9) | 0.038 | Chi-square test |
| Pae | 16 (26.7) | 22 (32.4) | 0.611 | Chi-square test |
| Kpn | 17 (28.3) | 21 (30.9) | 0.904 | Chi-square test |
| HRV | 8 (13.3) | 0 (0.0) | 0.002 | Fisher's exact test |
| IFA | 3 (5.0) | 5 (7.4) | 0.722 | Fisher's exact test |
| SARS-CoV-2 | 2 (3.3) | 3 (4.4) | 1 | Fisher's exact test |
| Cal | 20 (33.3) | 28 (41.2) | 0.464 | Chi-square test |
| Pji | 3 (5.0) | 3 (4.4) | 1 | Fisher's exact test |
| Lpn | 3 (5.0) | 0 (0.0) | 0.1 | Fisher's exact test |
| Aba | 13 (21.7) | 22 (32.4) | 0.248 | Chi-square test |
| Spn | 3 (5.0) | 6 (8.8) | 0.5 | Fisher's exact test |
| Hin+Spn | 2 (3.3) | 3 (4.4) | 1 | Fisher's exact test |
| Mca+Pji | 1 (1.7) | 1 (1.5) | 1 | Fisher's exact test |
| Sau+IFA | 2 (3.3) | 0 (0.0) | 0.218 | Fisher's exact test |
| Kpn+MRS-mecA | 7 (11.7) | 9 (13.2) | 1 | Chi-square test |
| Aba+Cal | 6 (10.0) | 14 (20.6) | 0.161 | Chi-square test |
| MRS-mecA+MTB | 13 (21.7) | 13 (19.1) | 0.891 | Chi-square test |
| Kpn+MRS-mecA+MTB | 6 (10.0) | 9 (13.2) | 0.77 | Chi-square test |
| Aba+MTB+Cal | 5 (8.3) | 9 (13.2) | 0.547 | Chi-square test |
| Aba+Kpn+MTB | 5 (8.3) | 7 (10.3) | 0.939 | Chi-square test |
| Aba+Kpn+MRS-mecA+MTB | 3 (5.0) | 5 (7.4) | 0.722 | Fisher's exact test |
| Aba+Kpn+MTB+Cal | 3 (5.0) | 4 (5.9) | 1 | Fisher's exact test |
| Kpn+MRS-mecA+MTB+Pae | 4 (6.7) | 1 (1.5) | 0.185 | Fisher's exact test |

**Supplementary Table 8** Selected Pathogen and Co-detection Features Stratified by ICU Length of Stay (Data Used for Figure 4A)

| Pathogen detection | <15, n(%) | ≥15, n(%) | *p*-value | Statistical test |
| --- | --- | --- | --- | --- |
| MTB | 45 (64.3) | 40 (66.7) | 0.921 | Chi-square test |
| Cal | 21 (30.0) | 29 (48.3) | 0.05 | Chi-square test |
| Pae | 21 (30.0) | 18 (30.0) | 1 | Chi-square test |
| Kpn | 21 (30.0) | 17 (28.3) | 0.988 | Chi-square test |
| Aba | 17 (24.3) | 18 (30.0) | 0.593 | Chi-square test |
| MRS-mecA+MTB | 12 (17.1) | 15 (25.0) | 0.377 | Chi-square test |
| Aba+Cal | 10 (14.3) | 10 (16.7) | 0.896 | Chi-square test |
| Kpn+MRS-mecA | 7 (10.0) | 9 (15.0) | 0.55 | Chi-square test |
| Kpn+MRS-mecA+MTB | 7 (10.0) | 8 (13.3) | 0.751 | Chi-square test |
| Aba+MTB+Cal | 9 (12.9) | 5 (8.3) | 0.585 | Chi-square test |
| Aba+Kpn+MTB | 7 (10.0) | 5 (8.3) | 0.981 | Chi-square test |
| Spn | 6 (8.6) | 4 (6.7) | 0.752 | Fisher's exact test |
| Aba+Kpn+MRS-mecA+MTB | 3 (4.3) | 5 (8.3) | 0.47 | Fisher's exact test |
| HRV | 5 (7.1) | 3 (5.0) | 0.725 | Fisher's exact test |
| IFA | 6 (8.6) | 2 (3.3) | 0.286 | Fisher's exact test |
| Aba+Kpn+MTB+Cal | 6 (8.6) | 1 (1.7) | 0.123 | Fisher's exact test |
| Pji | 4 (5.7) | 2 (3.3) | 0.686 | Fisher's exact test |
| SARS-CoV-2 | 2 (2.9) | 3 (5.0) | 0.662 | Fisher's exact test |
| Hin+Spn | 3 (4.3) | 2 (3.3) | 1 | Fisher's exact test |
| Kpn+MRS-mecA+MTB+Pae | 3 (4.3) | 2 (3.3) | 1 | Fisher's exact test |
| Lpn | 2 (2.9) | 1 (1.7) | 1 | Fisher's exact test |
| Mca+Pji | 1 (1.4) | 1 (1.7) | 1 | Fisher's exact test |
| Sau+IFA | 1 (1.4) | 1 (1.7) | 1 | Fisher's exact test |

**Supplementary Table 9** Sensitivity analyses of the association between pathogen detection burden and clinical outcomes

| Analysis | Exposure | Adjusted covariates | OR (95% CI) | *p*-value | Hazard Ratio (95% CI) | *p*-value |
| --- | --- | --- | --- | --- | --- | --- |
| Main | Total detection count | Age + APACHE-II Score + Prior anti-infective therapy within 7 days + Pre-ICU hospitalization days | 1.55 (1.14 - 2.16) | 0.007 | 1.42 (1.11 - 1.82) | 0.006 |
| S1 | Detection count excluding Cal | Age + APACHE-II Score + Prior anti-infective therapy within 7 days + Pre-ICU hospitalization days | 1.57 (1.12 - 2.24) | 0.010 | 1.48 (1.13 - 1.94) | 0.005 |
| S2 | Total detection count | Age + APACHE-II Score | 1.56 (1.15 - 2.17) | 0.006 | 1.42 (1.11 - 1.83) | 0.006 |
| S3 | Detection count excluding Cal | Age + APACHE-II Score | 1.58 (1.14 - 2.25) | 0.008 | 1.48 (1.13 - 1.94) | 0.004 |
| S4 | Total detection count | Age + APACHE-II Score + Immunocompromised status | 1.57 (1.16 - 2.20) | 0.005 | 1.48 (1.15 - 1.92) | 0.003 |
| S5 | Detection count excluding Cal | Age + APACHE-II Score + Immunocompromised status | 1.59 (1.14 - 2.27) | 0.007 | 1.53 (1.16 - 2.02) | 0.002 |
| S6 | Total detection count after excluding immunocompromised patients | Age + APACHE-II Score + Prior anti-infective therapy within 7 days + Pre-ICU hospitalization days | 1.57 (1.12 - 2.24) | 0.010 | 1.47 (1.12 - 1.93) | 0.006 |
| S7 | Detection count excluding Cal after excluding immunocompromised patients | Age + APACHE-II Score + Prior anti-infective therapy within 7 days + Pre-ICU hospitalization days | 1.54 (1.11 - 2.21) | 0.013 | 1.53 (1.16 - 2.02) | 0.002 |
| S8 | Detection count excluding pathogens with raw Ct >35 | Age + APACHE-II Score + Prior anti-infective therapy within 7 days + Pre-ICU hospitalization days | 1.48 (1.09 - 2.06) | 0.015 | 1.41 (1.09 - 1.82) | 0.009 |

**Supplementary Table 10** Exploratory summary of available raw Ct values for detected pathogen targets

| Pathogen detection | Positive detections with available raw Ct, n | Median raw Ct (IQR or range) | Median 2^(-ΔCt) (IQR or range) | Raw Ct ≤ 30, n (%) | 30 < raw Ct ≤ 35, n (%) | 35 < raw Ct ≤ 38, n (%) |
| --- | --- | --- | --- | --- | --- | --- |
| MTB | 52 | 25.67 (21.50 - 30.75) | 5.34E-03 (3.18E-04 - 9.89E-02) | 37 (71.2) | 11 (21.2) | 4 (7.7) |
| Pae | 17 | 19.83 (18.73 - 26.16) | 5.51E-01 (5.45E-03 - 2.33E+00) | 13 (76.5) | 3 (17.6) | 1 (5.9) |
| Kpn | 19 | 25.62 (22.55 - 33.02) | 7.44E-03 (2.18E-04 - 1.67E-01) | 13 (68.4) | 2 (10.5) | 4 (21.1) |
| Aba | 7 | 29.36 (25.98 - 31.39) | 1.53E-03 (2.41E-04 - 5.13E-03) | 5 (71.4) | 1 (14.3) | 1 (14.3) |
| MRS-mecA | 9 | 35.04 (29.50 - 35.23) | 2.36E-05 (3.28E-06 - 1.58E-02) | 3 (33.3) | 1 (11.1) | 5 (55.6) |
| Hin | 4 | 24.23 (18.50 - 29.35) | 3.14E-01 (8.82E-03 - 6.67E+01) | 3 (75.0) | 0 (0.0) | 1 (25.0) |
| Spn | 2 | 28.27 (21.97 - 34.57) | 6.72E-03 (1.85E-05 - 1.34E-02) | 1 (50.0) | 1 (50.0) | 0 (0.0) |
| HRV | 5 | 31.01 (28.26 - 31.18) | 9.90E-04 (8.74E-04 - 5.68E-03) | 2 (40.0) | 3 (60.0) | 0 (0.0) |
| IFA | 7 | 30.90 (27.01 - 32.73) | 6.10E-04 (3.23E-04 - 2.95E-03) | 3 (42.9) | 3 (42.9) | 1 (14.3) |
| SARS-CoV-2 | 5 | 30.49 (25.78 - 34.34) | 1.07E-02 (3.05E-05 - 2.78E-02) | 2 (40.0) | 2 (40.0) | 1 (20.0) |
| HCoV | 3 | 23.23 (19.02 - 30.98) | 3.58E-01 (3.28E-03 - 1.08E+00) | 2 (66.7) | 1 (33.3) | 0 (0.0) |
| PIV | 2 | 25.73 (18.81 - 32.66) | 4.06E-01 (9.12E-05 - 8.12E-01) | 1 (50.0) | 1 (50.0) | 0 (0.0) |
| RSV | 1 | 22.91 (22.91 - 22.91) | 1.26E-01 (1.26E-01 - 1.26E-01) | 1 (100.0) | 0 (0.0) | 0 (0.0) |
| Cal | 23 | 27.48 (23.54 - 31.62) | 6.62E-04 (3.70E-04 - 3.69E-02) | 15 (65.2) | 5 (21.7) | 3 (13.0) |
| Lpn | 1 | 36.35 (36.35 - 36.35) | 7.86E-08 (7.86E-08 - 7.86E-08) | 0 (0.0) | 0 (0.0) | 1 (100.0) |
| CP | 1 | 38.00 (38.00 - 38.00) | 6.98E-07 (6.98E-07 - 6.98E-07) | 0 (0.0) | 0 (0.0) | 1 (100.0) |
| Total pathogen detections | 158 | 27.30 (21.91 - 32.27) | 4.89E-03 (2.63E-04 - 1.17E-01) | 101 (63.9) | 34 (21.5) | 23 (14.6) |

Ct values were summarized only for positive detections with retrievable and target-assignable Ct values from the original amplification records. The denominator therefore represents positive detections with available Ct values rather than all positive detections for each target. For targets with fewer than four available Ct values, ranges rather than IQRs are reported where applicable. Ct-based summaries were considered exploratory because internal-control Ct values were not yet incorporated and Ct values from this single-tube multiplex PCR assay should not be interpreted as standardized quantitative pathogen-load measurements.Median 2^(-ΔCt) values (IQR or range) are additionally presented as exploratory relative signal measures derived from raw Ct values and should not be interpreted as standardized quantitative estimates of pathogen burden or as directly comparable across pathogen targets.

**Supplementary FIG 1** Sensitivity analyses of the association between pathogen detection burden and survival outcomes in critically ill patients. (A–D) Kaplan–Meier survival curves stratified by total pathogen detection burden in the overall cohort, including Cal in the detection count calculation: ≤1 vs >1 (A), ≤2 vs >2 (B), viral target positivity (C), and other pathogen target positivity (D). (E) ROC curve analysis for pathogen detection burden calculated after excluding Cal from the detection count, with an optimal cutoff value of 4.5 determined by the Youden index (AUC = 0.709). (F–I) Kaplan–Meier survival curves stratified by pathogen detection burden calculated after excluding Cal from the detection count using different thresholds: ≤1 vs >1 (F), ≤2 vs >2 (G), ≤3 vs >3 (H), and ≤4 vs >4 (I). For Kaplan–Meier panels, shaded areas indicate 95% confidence intervals and P values are from log-rank tests. Numbers at risk are shown below each plot.
